# Photobiomodulation restores blood–brain barrier integrity after hypoxia via endothelial von Willebrand factor modulation in a humanised tricellular transwell model

**DOI:** 10.1113/JP291064

**Published:** 2026-04-11

**Authors:** Mirriam Domocos, Denis E. Bragin, Nagesh C. Shanbhag, Luise Schlotterose, Mootaz M. Salman

**Affiliations:** ^1^ Department of Physiology, Anatomy, and Genetics University of Oxford Oxford UK; ^2^ British Heart Foundation (BHF) Oxford Centre of Research Excellence University of Oxford Oxford UK; ^3^ Kavli Institute for NanoScience Discovery University of Oxford Oxford UK; ^4^ British Heart Foundation (BHF) – UK Dementia Research Institute (UK DRI) Centre for Vascular Dementia Research University of Oxford Oxford UK; ^5^ Lovelace Biomedical Research Institute Albuquerque NM USA; ^6^ Department of Neurology University of New Mexico School of Medicine Albuquerque NM USA; ^7^ Department of Cell and Molecular Physiology New York Medical College Valhalla NY USA; ^8^ Meditech Foundation Cali Colombia

**Keywords:** blood–brain barrier (BBB), endothelial dysfunction, hypoxia, *in vitro* models, photobiomodulation, von Willebrand factor (vWF)

## Abstract

**Abstract:**

A functional blood–brain barrier (BBB) is essential for CNS homeostasis, and its disruption is an early feature of both acute brain injury and chronic neurodegenerative disorders. Hypoxia induces BBB breakdown by triggering endothelial dysfunction, oxidative stress, metabolic dysregulation and thrombo‐inflammatory signalling that compromise barrier integrity. However, strategies that restore BBB function remain limited. Here, we investigated whether photobiomodulation (PBM), a non‐invasive light therapy, can rescue BBB dysfunction following acute hypoxic stress. Using a multicellular *in vitro* BBB model comprising immortalised human brain microvascular endothelial cells, pericytes and astrocytes, we induced hypoxic injury (6 h, 1% O_2_) and applied three PBM treatments during recovery. Hypoxia significantly reduced transendothelial electrical resistance (TEER), whereas PBM restored barrier function in endothelial monocultures and tri‐cultures. Endothelial cells exhibited the most pronounced hypoxic response, characterised by increased expression of hypoxia‐inducible factor‐1α (HIF‐1α), plasminogen activator inhibitor‐1 and von Willebrand factor (vWF), all attenuated by PBM. Importantly, small interfering RNA‐mediated knockdown of vWF partially recapitulated PBM‐induced restoration of barrier integrity, identifying endothelial vWF as a mediator of recovery. PBM also reduced reactive oxygen species in hypoxic astrocytes and pericytes, indicating co‐ordinated multicellular modulation. Together, these findings demonstrate that PBM restores BBB integrity following hypoxic insult by modulating endothelial thrombo‐inflammatory signalling at the same time as reducing oxidative stress in glial cells. Rather than acting as a non‐specific cytoprotective stimulus, PBM engages molecular pathways linked to endothelial activation. This work establishes a mechanistically informed platform for investigating BBB repair and highlights PBM as a strategy to protect vascular integrity in hypoxia‐associated neurological disorders.

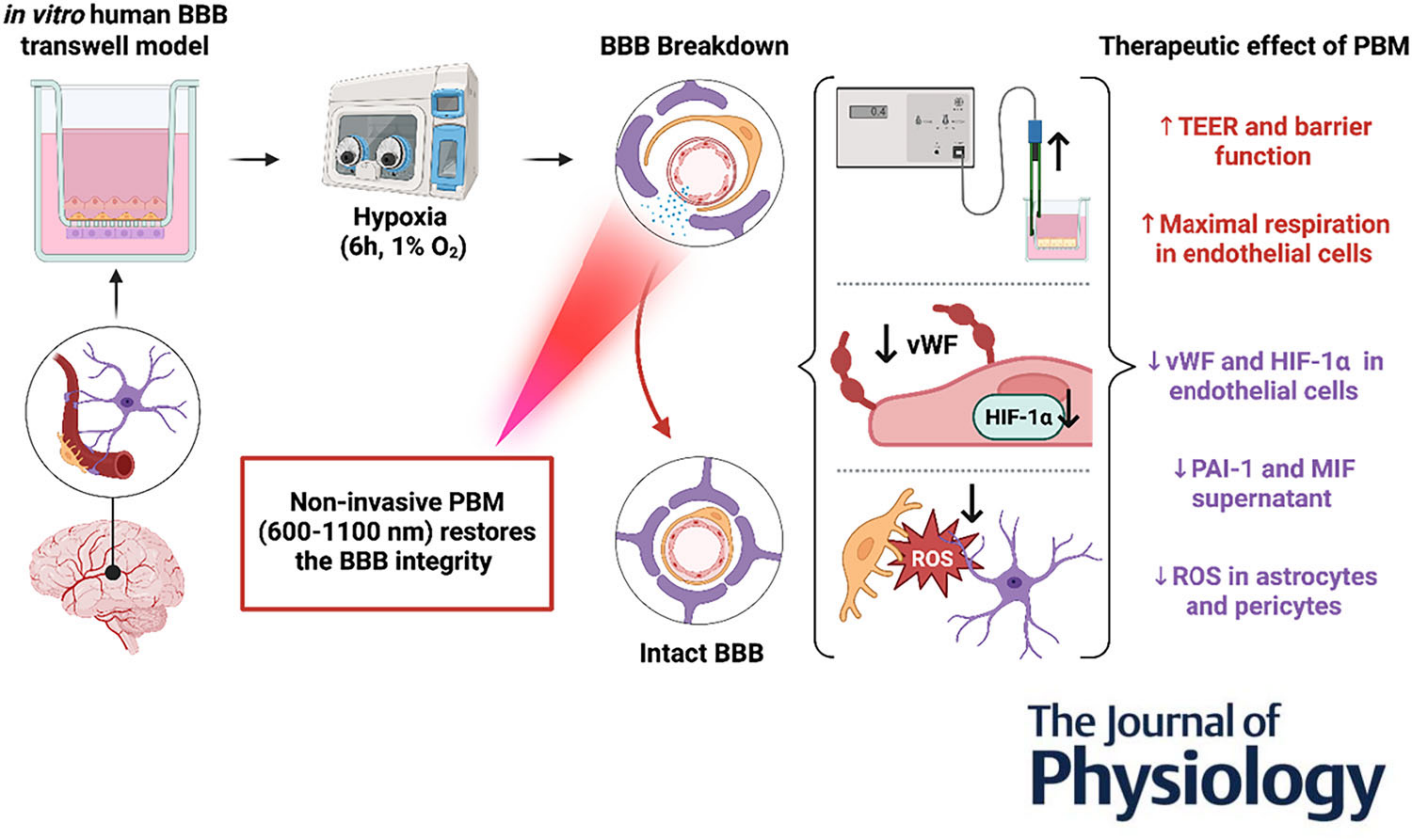

**Key points:**

- Hypoxia is a major driver of blood–brain barrier (BBB) dysfunction, yet there are currently no targeted therapies that directly restore barrier integrity.
- Photobiomodulation (PBM) is a non‐invasive low‐level light intervention known to facilitate mitochondrial function and cellular stress responses.
- In a human *in vitro* BBB model, repeated PBM treatment restored transendothelial electrical resistance (TEER) 24 and 48 h after hypoxic injury, with endothelial rescue linked to downregulation of von Willebrand factor (vWF).
- PBM modulated oxidative stress, hypoxia signalling and thrombo‐inflammatory pathways across endothelial cells, astrocytes and pericytes.
- These findings support PBM‐driven modulation of endothelial signalling as a potential strategy to restore BBB integrity in hypoxia‐associated neurological conditions.

## Graphical abstract

**Abstract figure legend** Photobiomodulation restores blood–brain barrier (BBB) integrity following hypoxic stress. A multicellular human BBB model comprising brain microvascular endothelial cells, astrocytes and pericytes was established in a transwell system and subjected to acute hypoxia. Photobiomodulation (PBM) was applied during the recovery phase. PBM restored barrier integrity, evidenced by increased transendothelial electrical resistance (TEER) and enhanced maximal mitochondrial respiration in endothelial cells. Mechanistically, PBM attenuated hypoxia‐induced endothelial activation, reducing hypoxia‐inducible factor‐1 alpha (HIF‐1α) and von Willebrand factor (vWF) expression, and decreased secretion of thrombo‐inflammatory mediators, including plasminogen activator inhibitor‐1 (PAI‐1) and migration inhibitory factor (MIF). PBM also reduced reactive oxygen species (ROS) levels in astrocytes and pericytes, indicating co‐ordinated multicellular modulation of oxidative stress. Together, these findings identify endothelial thrombo‐inflammatory signalling and glial oxidative stress as key pathways through which PBM restores BBB function following hypoxic injury.

## Introduction

The blood–brain barrier (BBB) is a highly specialised and dynamically regulated multicellular interface that preserves CNS integrity by controlling nutrient and oxygen supply, co‐ordinating metabolic waste clearance, and preventing the entry of potentially neurotoxic circulating factors (Alhadidi et al., 2024). The brain endothelial cells form a continuous monolayer lining the blood vessel, distinguished from peripheral endothelial cells by high expression of tight junction proteins, most importantly zonula occludens‐1 (ZO‐1), claudin‐5 and occludin (Rust et al., 2025; Wu et al., 2023). Wrapped around endothelial cells are pericytes that provide structural support and also maintain the BBB via expression of the platelet‐derived growth factor receptor beta (PDGFRβ). Brain pericytes on larger vessels express higher levels of alpha smooth muscle actin (αSMA) to regulate the blood flow and oxygenation of the CNS (Liebner et al., 2018; Rust et al., 2025). The astrocytes complete the BBB by ‘grabbing’ the vessel with their flattened endfeet, allowing close communication between the vasculature and neurons. An essential protein of the endfeet structure is the water channel aquaporin‐4 (AQP4) that facilitates bidirectional water movement through the brain (Salman et al., 2022; Verkman et al., 2013). Given the importance of the BBB in brain homeostasis, BBB damage is associated with a variety of disease, including dementias and stroke, and leads to the structural breakdown of tight junctions and to the loss of endothelial cells, pericytes and astrocytes. This disrupts normal nutrient transport and increases BBB permeability leading to a leaky barrier, and triggers inflammatory responses that cause neuronal damage, impaired waste clearance, and brain and cell death (Ayyappan et al., 2026; Profaci et al., 2020). Hypoxia is a common source of BBB dysfunction where low oxygenation stabilises hypoxia‐inducible factor‐1 alpha (HIF‐1α), which translocates to the cell nucleus and facilitates inflammatory responses, metabolic reprogramming and vascular remodelling. In addition, hypoxia leads to oxidative stress, characterised by mitochondrial dysfunction and upregulation of reactive oxygen species (ROS) (Kim et al., 2006; Merelli et al., 2018; Salman et al., 2017; Schlotterose et al., 2024). In endothelial cells, the platelet adhesion molecule von Willebrand factor (vWF) is upregulated, contributing to leukocyte infiltration and a decrease in tight junction proteins (Kawecki et al., 2017). Pericytes and astrocytes detach from the basal membrane, leaving the BBB unsupported and thus downregulating key cell markers linked to BBB function. Overall, hypoxic‐ and HIF‐1α‐mediated mechanisms impair the barrier integrity and with a lack of therapeutic interventions this leaves a vicious cycle of BBB dysfunction and disease progression (Engelhardt et al., 2014).

Photobiomodulation (PBM) is a non‐invasive and US Food and Drug Administration (FDA)‐approved low‐level light therapy that has been widely applied in dermatology, sports injury, pain management and tissue repair. PBM typically employs red and near‐infrared wavelengths ranging from 600 to 1100 nm, which are absorbed by mitochondrial chromophores including cytochrome *c* oxidase (CCO), the terminal enzyme of the respiratory chain and a proposed primary photoacceptor for PBM (Huang et al., 2024; Karu, 1999). Light absorption at CCO enhances mitochondrial respiration and oxidative phosphorylation, leading to increased ATP synthesis and transient modulation of mitochondrial membrane potential (Rojas & Gonzalez‐Lima, 2011; Wong‐Riley et al., 2005). These primary bioenergetic changes are accompanied by alterations in ROS signalling and activation of redox‐sensitive transcriptional programmes that regulate cellular stress responses, inflammation and survival pathways (Hamblin, 2017; Salehpour et al., 2018). Additional mechanisms have been proposed, including nitric oxide photodissociation from mitochondrial respiratory complexes, which may further enhance electron transport and mitochondrial activity (Hamblin, 2018; Poyton & Ball, 2011). Consistent with these mechanisms, PBM has been shown to modulate oxygen free radicals and cellular redox balance (Yan et al., 2025). Collectively, mitochondrial CCO engagement and downstream redox signalling provide a biologically plausible route by which PBM can influence endothelial metabolism, oxidative stress and inflammatory mediators relevant to BBB integrity.

Beyond its metabolic effects, PBM has demonstrated beneficial outcomes in neurological disease models. In APP/PS1 transgenic mice, PBM improves cognitive defects and anxiety at the same time as increasing expression of tight junction proteins including ZO‐1, claudin‐5 and occludin, thus enhancing BBB integrity (Ma et al., 2025). The clinical relevance of PBM has also been explored in vascular contexts, where it has been reported to improve endothelial recovery following coronary intervention and maintain endothelial homeostasis through modulation of endothelial nitric oxide and transforming growth factor‐β signalling (Colombo et al., 2021). Early clinical and translational studies applying PBM in neurological disorders further support its feasibility and potential therapeutic benefit (Berman et al., 2017; Chao, 2019; Salehpour & Hamblin, 2020). Despite this growing interest, the cell‐specific mechanisms through which PBM acts on the BBB remain poorly defined, and it is unclear whether PBM can directly restore barrier integrity following hypoxic injury.

To address this gap, we hypothesised that PBM restores BBB integrity after hypoxic stress through defined endothelial thrombo‐inflammatory signalling pathways rather than through non‐specific cytoprotection. To test this, we investigated the therapeutic impact of PBM on BBB function under hypoxic conditions and dissected the intrinsic responses of individual cellular components of the barrier. We employed a controlled *in vitro* BBB model consisting of immortalised human brain microvascular endothelial cells co‐cultured with human astrocytes and vascular pericytes in a transwell system. This reductionist yet physiologically informed platform enabled precise interrogation of barrier function and facilitated downstream mechanistic assays to define cell‐type‐specific responses.

Our findings demonstrate that three sessions comprising 5 min of PBM irradiation significantly restored barrier function in both endothelial monocultures and BBB tri‐cultures following hypoxic insult (6 h at 1% O_2_). This functional recovery was associated with downregulation of hypoxia‐induced vWF in endothelial cells, attenuation of HIF‐1α expression and enhancement of maximal mitochondrial respiratory capacity, indicating improved metabolic resilience. PBM also reduced ROS levels in hypoxic astrocytes and pericytes, highlighting co‐ordinated multicellular responses. Cytokine and chemokine profiling further revealed modulation of thrombo‐inflammatory pathways consistent with a protective anti‐thrombotic milieu. Collectively, these findings reveal previously unrecognised mechanisms by which PBM restores BBB function after hypoxic injury and highlight its potential as a therapeutic strategy for cerebrovascular dysfunction.

## Methods

### Cell culture lines

The cell lines used were purchased from Innoprot: human brain microvascular endothelial cells (HBMECs) (P10361‐IM; Innoprot, Derio, Spain), human astrocytes (HAs) (P10251‐IM; Innoprot) and human brain vascular pericytes (HVPCs) (P10363‐IM; Innoprot). The cell lines were immortalised via the SV40 antigen T. Cells were used up to passage number 20 and maintained at 37°C and 5% CO_2_ in the appropriate medium purchased from Innoprot, with a full medium change every 2 days: endothelial cell medium (EM) (P60104; Innoprot), astrocyte medium (AM) (P60101; Innoprot) or pericyte medium‐PLUS (PM) (P60121‐Plus; Innoprot).

### Culture for transwell models

Cell‐to‐cell contact models were established to facilitate communication between cells on Millicell® 24‐well hanging inserts (PTSP24H48; Millipore, Burlington, MA, USA) with a pore size of 3 µm and area of 0.3 cm^2^. On day 1, the basal sides of the inserts were inverted, resting inside the cover plate lid, and coated with 50 µL of poly‐l‐lysine (PLL) (2 µg cm^−2^) (P4705; Sigma‐Aldrich, St Louis, MO, USA). A cover plate lid was placed on top to distribute the coating and the plate was incubated at room temperature (RT) for 1 h. PLL coatings were washed with phosphate‐buffered saline (10010023; Gibco, Waltham, MA, USA) and for models with HAs, 20  µL of HA suspension (1.5 × 10^4^ cells) was evenly pipetted on the basal site of the membrane. The bottom of the well plate was inverted without damaging any inserts, and the cells were incubated at 37°C for 2 h. The entire plate was flipped back with the basal side facing down, and each well was topped up with 1.3 mL of AM.

On day 2, the apical sides of the inserts were coated with 200 µL of Geltrex (A1413302; Thermo Fisher Scientific, Waltham, MA, USA) and incubated at 37°C for 1 h. Geltrex was selected as the most suitable apical coating compared to PLL and Matrigel (Corning Inc., Corning, NY, USA). Transendothelial electrical resistance (TEER) measurements revealed that Geltrex facilitated the highest barrier function in HBMEC monocultures (*P* = 0.0045) and in co‐cultures with HAs (*P* = 0.0207). Geltrex was fully aspirated and, for cultures with HVPCs, 100 µL of HVPC suspension (2.5 × 10^4^ cells) was pipetted into the insert, followed by incubation for 10 min. For models with HVPCs and HBMECs co‐cultured at the apical site, 100 µL of HBMEC suspension containing 7.5 × 10^4^ cells was used, for a total of 1.0 × 10^5^ cells seeded within the insert. Then, 500 µL of EM:PM media was added at 1:1. For models with only HBMECs on the apical site, 100 µL of HBMEC suspension with 1.0 × 10^5^ cells was pipetted and 500 µL of EM was added. The cultures were incubated at 37°C until day 8 when conditioning took place.

### Experimental groups

**Figure 1:**
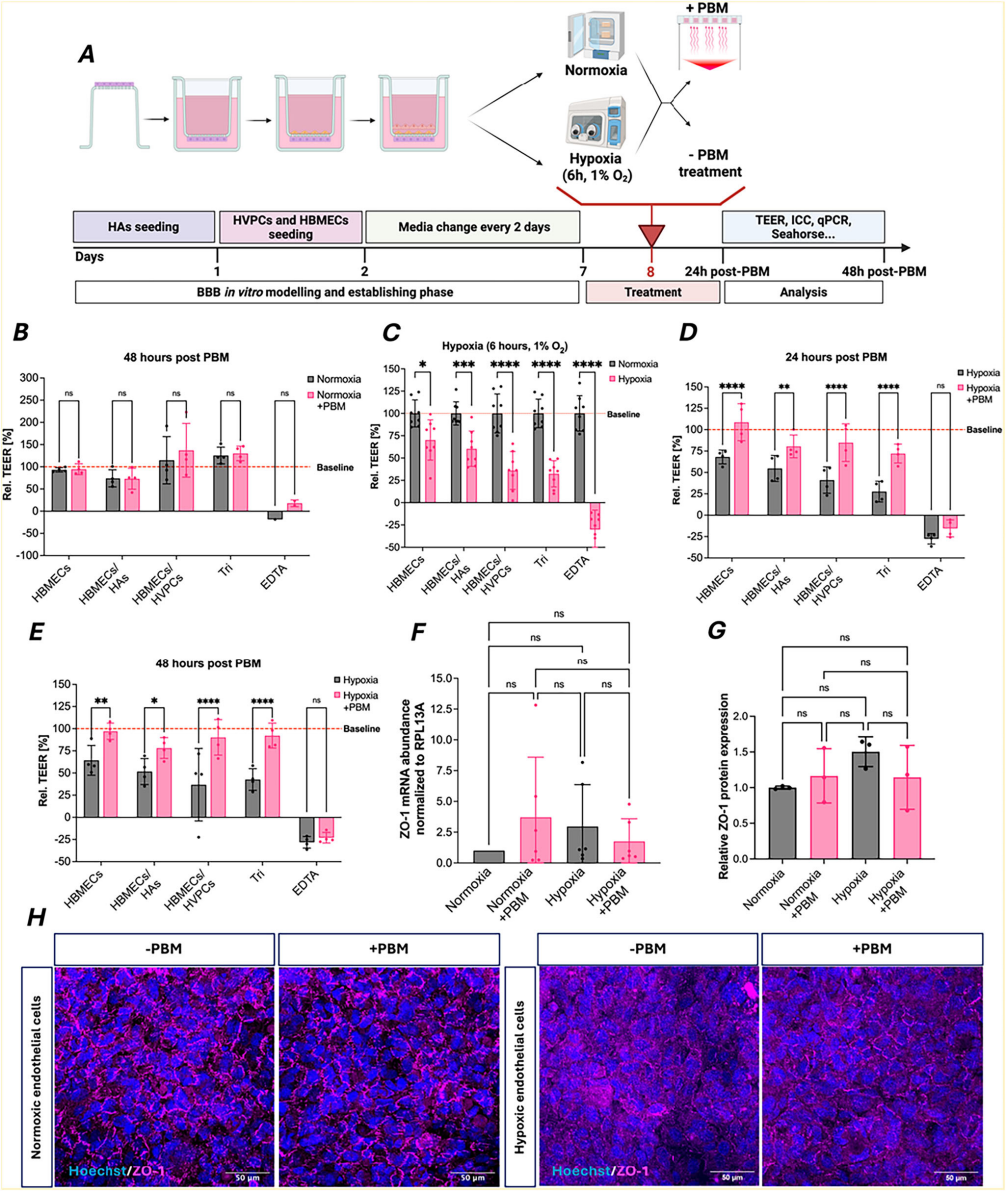
PBM restores endothelial junctional integrity and barrier function following hypoxic injury ***A***, experimental workflow illustrating the transwell‐based BBB model, hypoxic exposure (6 h, 1% O_2_), PBM treatment schedule, and downstream functional and molecular analyses (created with BioRender). ***B***–***E***, TEER expressed as relative change (%) from baseline. ***B***, normoxic controls at 48 h. ***C***, TEER immediately following hypoxia. ***D***, TEER at 24 h post‐hypoxia. ***E***, TEER at 48 h post‐hypoxia. PBM significantly restored endothelial barrier resistance under hypoxic conditions. ***F***, ZO‐1 mRNA expression in HBMECs at 48 h, normalised to RPL13A. ***G***, quantification of ZO‐1 protein levels relative to control, measured as ZO‐1‐positive area normalised to Hoechst nuclear area. ***H***, representative ICC images showing ZO‐1 (magenta) and nuclei (Hoechst, blue) in normoxic and hypoxic HBMECs with and without PBM. Statistical comparisons of means were performed using two‐way ANOVA with Tukey's multiple comparisons test (*P < 0.05, **P < 0.01 and ***P < 0.001); *N* = 3–5 independent biological replicates as independent cell cultures. Data are presented as the mean ± SD.

On day 8, transwell models were either kept as an untreated control group (1) normoxia, or exposed to (2) PBM treatment and (3) hypoxia (6 h at 1% O_2_), or (4) hypoxia followed by PBM. The topwrap TW‐03B (ProNeuroLight, LLC, Phoenix, AZ, USA) was used to irradiate the cultures with red and infrared light in three sessions either under normoxia or after hypoxia. The plate was positioned 10 cm above the flat‐laid topwrap, and the transwell models were irradiated for 5 min. Between sessions, plates were incubated at 37°C for a recovery period of 1 h. PBM treatments were performed with the main light turned off, and the device placed away from the window. An EDTA‐treated (50 mm) (15575‐020; Invitrogen) tri‐culture acted as a negative control of barrier breakdown for all four groups. Functional and quantitative analyses were carried out 24 and 48 h after conditioning on day 9 or 10 (Fig. 1*A*).

The multi‐wavelength topwrap TW‐03B device consists of two dual LED chips that simultaneously emit red and infrared light at coupled wavelengths of 635 + 904 and 810 + 1064 nm. Based on manufacturer specifications and the experimental geometry used in this study, the estimated total irradiance at the culture plane (at 10 cm of distance) was approximately 111.5 mW/cm^2^. This corresponds to an estimated fluence at the culture plane of approximately 33.5 J/cm^2^ per treatment session and approximately 100 J/cm^2^ across the three treatment sessions. These irradiance and fluence values were derived from device specifications and experimental geometry, with the plate covering only half of the topwrap, and were not directly measured at the sample plane, and should therefore be considered approximate. Further details are provided in the Supporting Information file ‘Estimated photobiomodulation dosimetry under experimental conditions’.

[Correction added on 27 June 2026, after first online publication: Five sentences detailing additional photobiomodulation dosimetry parameters have been added to the Methods section.]

### TEER measurements

TEER was calculated based on the resistance measured with the EVOM Manual Meter (EVM‐MT‐03‐02; World Precision Instruments) according to accirdance with the manufacturer's instruction for handling and measuring. Electrodes were positioned using the electrode placement frame, with the shorter electrode in the insert and the longer outside the transwell. Per transwell, three individual resistance measurements were recorded after being referenced to the blank. The TEER is the product of the membrane's area (0.3 cm^2^) and the referenced resistance. TEER was then averaged and taken relative to the baseline TEER to calculate the change in relative TEER. Measurements were taken in four biological replicates.

### ToxiLight cytotoxicity assay

The ToxiLight non‐destructive cytotoxicity bioassay kit (LT17‐217; Lonza, Basel, Switzerland) was used according to accordance with the manufacturers protocol and carried out for the hypoxia groups after 48 h. Cell supernatant from the insert (20 µL) was transferred to a white 96‐well plate with clear bottoms with two technical replicates per sample. Then, 100 µL of the reconstituted adenylate kinase detection reagent was added to each well and incubated for 5 min. The luminescence was measured with the CLARIOstar PLUS microplate reader (BMG Labtech, Ortenberg, Germany) and the relative average luminescence (RLU) calculated by dividing the luminescence of samples by that of their respecive cell‐free media. To calculate the relative luminescence the end‐point RLU was divided by the baseline RLU. Measurements were taken in four biological replicates with every two technical replicates.

### AlamarBlue cell viability assay

The AlamarBlue reagent assay (DAL1025; Invitrogen) was used to detect cell viability in normoxia and hypoxia groups at baseline (day 8) and after 48 h. A proportion (10%) of the apical and basal volumes of the alamarBlue dye was added to the appropriate compartments and incubated at 37°C for 2 h. The sample was pipetted into a black 96‐well microplate to a volume of 10% of the apical and basal volumes. The fluorescence was measured at excitation/emission of 495/519 nm with the CLARIOstar PLUS microplate reader and the relative fluorescence signal was calculated by taking the average of the fluorescence from both compartments, after subtracting the background signal from the blank (the media), relative to the baseline fluorescence signal before any conditioning. Measurements were taken in four biological replicates.

### Immunocytochemistry (ICC)

Transwell cultures were fixed with 4% paraformaldehyde (#28908; Thermo Fisher Scientific) in both compartments and washed twice with phosphate‐buffered saline for 5 min. Cells were permeabilised with 0.3% Triton X‐100 (HFH10; Invitrogen) for 10 min at RT and washed twice again. Blocking was performed with 0.5% bovine serum albumin (A8806‐1G; Sigma‐Aldrich) and 0.5% glycine (G8898‐500G; Sigma‐Aldrich) for 1 h at RT. Primary antibodies (Table 1) were diluted appropriately in blocking solution (BS), and cells were incubated in both compartments at 4°C overnight. After washing twice for 10 min, cells were incubated with secondary antibodies (Table 1) and Hoechst (H2569; Invitrogen) (0.8 µL per 1 mL) diluted in BS for 1.5 h. Cells were washed twice for 5 min, and membranes were cut out with a scalpel and mounted with Fluoromount (#345789; Millipore). Cells were imaged with the 3i Marianas Confocal Microscope (Intelligent Imaging Innovations) at 63≥ and analysed via ImageJ, version 1.54p (National Institutes of Health, Bethesda, MD, USA) with the Fiji extension and Java 21.0.7 (64‐bit). Analysis was performed on three biological replicates, with three representative images per replicate.

**Table 1:** Primary and secondary antibodies used for staining of endothelial cells, astrocytes and pericytes.

Antibody	Host	Dilution	Catalogue number	Supplier
ZO‐1	Rat	1:200	15652	Cell Signaling
vWF	Sheep	1:400	ab11713	Abcam
AQP4	Rabbit	1:200	ab128906	Abcam
GFAP	Rat	1:500	13‐0300	Invitrogen
PDGFRb	Rabbit	1:200	MA5‐15143	Invitrogen
HIF‐1α	Mouse	1:200	MA1‐516	Thermo Scientific
Actinred 555	–	2 drops per mL	R37112	Life Technologies Ltd
Anti‐sheep 647	Donkey	1:1000	A21448; LOT: 2720396	Invitrogen
Anti‐rat 488	Donkey	1:1000	A21208; LOT: 2668657	Invitrogen
Anti‐mouse 555	Donkey	1:1000	A31570; LOT: 3034154	Invitrogen
Anti‐rabbit 647	Donkey	1:1000	A31573; LOT: 2997084	Invitrogen
Anti‐mouse 488	Donkey	1:1000	A2120; LOT: 3006753	Invitrogen

### RNA extraction and quantitative real‐time PCR analysis

HBMECs, HVPCs and HAs were plated in 12‐well plates (3.0 × 10^5^ cells), cultured to a confluency of 80% and then conditioned for each group. After 48 h, total RNA was extracted with TRIzol (R2050‐1‐200; Zymo, Irvine, CA, USA) and purified with the Direct‐zol RNA MicroPrep kit (R2062; Zymo). RNA was quantified with the NanoDrop One/OneC spectrophotometer (Thermo Fisher Scientific). To generate 20 µL of cDNA (500 ng), 1 µL of Random Primers (1:20) (48190011; Invitrogen) and 1 µL of 10 mm dNTP mix (18427089; Invitrogen) were added to each diluted RNA sample for a final volume of 13 µL. The mixture was heated at 65°C for 5 min and then cooled to 4°C for at least 1 min. The reverse transcription was accomplished using 1 µL of SuperScript III RT (200 U µL^−1^), 4 µL of 5x First Strand Buffer, 1 µL of 0.1 m DTT, included in the SuperScript III RT kit (18080093l Invitrogen), and 1 µL of RnaseOUT Recombinant Ribonuclease Inhibitor (40 U µL^−1^) (10777019; Invitrogen) under the conditions: 65°C for 5 min, 55°C for 60 min and 70°C for 15 min. One quantitative PCR (qPCR) reaction (20 µL) contained 10 µL of 1x Fast SYBR Green Master Mix (4385612; Applied Biosystems, Waltham, MA, USA), 1 µL of each the relevant forward and reverse primers (0.2 µm) (Table 2), 5 µL of cDNA template (1:10) and 3 µL dH_2_O. *RPL13A* was used as the housekeeping gene. Reactions were run with the QuantStudio 3 Real‐time PCR System (A28567; Applied Biosystems) and the qPCR data was analysed following the 2^−ΔΔCT^ method normalising to the untreated normoxic cells as the control. The assay was measured in four to six biological replicates.

**Table 2:** Forward and reverse sequences for all primers used in 5′ to 3′

Genes	Forward primer (5′‐ to 3′)	Reverse primer (5′‐ to 3′)
*RPL13A*	GGTCCTGGTGCTTGATGGTC	GGCCCAGCAGTACCTGTTTA
*ZO‐1*	CCGCGGAGTTTCGGGTC	TCCTCCATTGCTGTGCTCTTG
*vWF*	GCTCATGCAACATCTCCTCTG	ACTCACACAAAGTCTTCTCACA
*PDGFRb*	CCACACTCCTTGCCCTTT	CACAGACTCAATCACCTTCCA
*α‐SMA*	AAAAGACAGCTACGTGGGTGA	GCCATGTTCTATCGGGTACTTC
*Rgs5*	GAGTTGAAGAGCAAAACCAAGTG	CTGAAGATATCCAGACAGTGCT
*AQP4*	AGGCAATGAGAGCTGCAC	AAGCCACCATGATGTTCTCT
*GFAP*	CCTGCAGATTCGAGAAACCA	CCTGCTTGGACTCCTTAATGAC
*HIF‐1α*	ATCACCCTCTTCGTCGCTTC	GGAAAGGCAAGTCCAGAGGT

### Small interfering RNA (siRNA) transfection and knockdown

To knock down vWF, HBMECs (5 × 10^5^ cells) were plated in Geltrex‐coated six‐well plates and cultured for 2 days. Pre‐annealed and pre‐designed siRNA (AM16708; Life Technologies Ltd, Carlsbad, CA, USA) was used to target vWF in accordance with the manufacturers protocol. For one well, 9 µL of Lipofectamine RNAiMAX Reagent (13778‐075; Life Technologies Ltd) was mixed with 150 µL of Opti‐MEM Medium (31985062; Gibco). Then, 3 µL of reconstituted siRNA (Working solution: 10 µm) was added to 150 µL of Opti‐MEM Medium. The dilutions were then mixed 1:1 and incubated at RT for 5 min. Next, 250 µL of the siRNA‐lipid complex was added to HBMECs, and the cells were incubated at 37°C for 48 h. To investigate the involvement of vWF in the pathway, four biological replicates of siRNA‐treated HBMECs were cultured. To confirm the knockdown of vWF, 5 × 10^5^ treated HBMECs were centrifuged and the pellet was subjected to further qPCR analysis.

### ROS‐Glo H2O2 assay

The ROS‐Glo H_2_O_2_ assay (G8820; Promega, Madison, WI, USA) was used to analyse the hydrogen peroxide (H_2_O_2_) production for normoxia and hypoxia groups at baseline, after one, two and three irradiations of PBM, and after 48 h in accordance with the manufacturer's protocol. Cells were plated in 96‐well plates (1.5 × 10^5^ cells), cultured to a confluency of 80% and then conditioned for each group. 20 µL of the prepared H_2_O_2 _Substrate solution was added and the cells were incubated at 37°C for 1 h. Next, 100 µL of the supernatant was transferred to a white 96‐well plate and 100 µL of the prepared ROS‐Glo detection solution was added. After incubating at RT for 20 min, the luminescence was measured with the CLARIOstar PLUS microplate reader. Measurements were taken in four biological replicates with three technical replicates.

### Seahorse real‐time cell metabolic analysis

HBMECs, HVPCs and HAs were plated on a Seahorse XFe 96 cell culture plate (3.0 × 10^5^ cells) (103794‐100; Agilent, Santa Clara, CA, USA) and the assay was carried out for normoxia and hypoxia groups after 24 and 48 h in accordance with the manufacturer's protocol. Cells were cultured for 2 days before being conditioned for each group. On the day of the assay, cells were washed twice with 50 µL of the prepared assay media (40 mL of XF base medium (103575‐100; Agilent), 0.0728 g of d‐glucose (10 mm) (G7021; Sigma‐Aldrich), 0.00448 g of sodium pyruvate (1 mm) (P5280‐25G; Sigma‐Aldrich) and 400 µL of l‐glutamine (2 mm) (25030024; Life Technologies Ltd). Assay media (175 µL) was added and cells were incubated at 37°C in a CO_2_‐free incubator for 1 h. After calibration of the Seahorse XFe 96 Extracellular Flux Analyzer (Agilent), three baseline recordings were made, followed by five after treatment with 1 µm Oligomycin (O4876‐5MG; Sigma‐Aldrich), three after treatment with 1 µm carbonyl cyanide‐4 (trifluoromethoxy) phenylhydrazone (FCCP) (Sigma‐Aldrich; C2920‐10MG) injection, three after treatment with 0.5 µm Rotenone/Antimycin A (R/A) (Sigma‐Aldrich: R8875‐1G;A867a) injection and five after treatment with 50 mm 2‐deoxy‐glucose (2‐DG) (Sigma‐Aldrich; D8375‐10MG) treatment. The oxygen consumption rate (OCR) and extracellular acidification rate (ECAR) were normalised to each well's total protein count quantified using the Pierce BCA protein assay kit (#23225; Thermo Fisher Scientific). Measurements were taken in four biological replicates with ten technical replicates.

### Proteome Profiler Human Cytokine Array

The relative expression levels of 36 cytokines, chemokines and acute phase proteins associated with inflammation were detected using the Proteome Profiler Human Cytokine Array Kit (ARY005B; R&D Systems, Minneapolis, MN, USA) under normoxia and hypoxia groups after 48 h in accordance with the manufacturers protocol. Membranes were blocked at RT for 1 h and incubated at 4°C overnight with the sample mixture containing 1 mL of cell‐free supernatant taken from tri‐cultures of each group, 500 µL of assay buffer 4 and 15 µL of human cytokine array detection antibody cocktail. After washing three times, 2 mL of streptavidin‐horseradish peroxidase (dilution 1:2000) was added and incubated at 25°C for 30 min on a shaker. Chemi reagents 1 and 2 were mixed 1:1 and then 1 mL was pipetted onto washed membranes before incubation RT for 1 min. The membranes were imaged with the iBright Imager 1500 (Invitrogen) auto‐exposure system and the mean intensity for each dot was quantified with Image J, version 1.54p with the Fiji extension. Measurements were taken in one biological replicate with two technical replicates.

### Statistical analysis

Statistical analyses were performed using Prism, version 10.0 (GraphPad Software Inc., San Diego, CA, USA). Data are presented as mean ± SD, based on a minimum of three independent biological replicates per condition. Comparisons between two independent groups were analysed using a two‐tailed unpaired Student's *t*‐test (with equal variance assumed unless variance testing indicated otherwise). For comparisons involving more than two groups with one independent variable, defined as the condition as used for the ROS levels, one‐way ANOVA followed by Tukey's *post hoc* test was used. When comparing multiple groups with two or more independent variables defined as the oxygenation and treatment, two‐way ANOVA followed by Tukey's multiple comparisons test was used. *P* < 0.05 was considered statistically significant.

## Results

### PBM restores hypoxia‐induced barrier dysfunction in optimised *in vitro* BBB models

TEER measurements provide a direct, quantitative readout of barrier function and are widely used to assess the integrity and tightness of the BBB. Figure 1*A* is a schematic representation of the workflow starting at day 1 and 2 with cell seeding followed by the appropriate treatment or conditioning on day 8. To exclude thermal artefacts, medium temperature was monitored continuously during PBM exposure. Only a minimal increase of 0.85°C was detected, confirming that the treatment did not produce significant heating. PBM irradiation did not affect the TEER for normoxia groups at 48 h (Fig. 1*B*); thus, cultures were exposed to hypoxia to explore PBM's potential therapeutic effect in disease. Hypoxia conditions (6 h at 1% O_2_) were selected based on prior literature (Chaudary et al., 2020; Hayashi et al., 2004) and showed a significant decrease in TEER of around 30–70% under hypoxia (*P* = 0.0118, *P* = 0.0004 and *P* < 0.0001) across all cultures compared to normoxic cultures (Fig. 1*C*). After 24 h, PBM led to a significant recovery close to the baseline levels in TEER after hypoxia for HBMEC monocultures (*P* < 0.0001; 108.7% ± 10.82), co‐cultured with HAs (*P* = 0.0037; 80.47% ± 6.671), co‐cultured with HVPCs (*P* < 0.0001; 84.83% ± 10.97) and BBB tri‐cultures (*P* < 0.0001; 72.15% ± 5.524) compared to non‐PBM‐treated cultures (68.10% ± 4.048, 54.67% ± 7.613, 41.06% ± 7.703 and 27.63% ± 6.057, respectively) (Fig. 1*D*). This phenomenon was also observed 48 h after hypoxia with HBMEC monocultures (*P* = 0.003; 97.06% ± 4.594), co‐cultured with HAs (*P* = 0.0222; 78.22% ± 5.861), co‐cultured with HVPCs (*P* < 0.0001; 90.21% ± 9.986) and BBB tri‐cultures (*P* < 0.0001; 92.21% ± 7.029) showing a restored TEER compared to non‐PBM‐treated cultures (64.33% ± 8.411, 51.66% ± 7.344, 36.83% ± 20.46 and 42.71% ± 6.108, respectively) (Fig. 1*E*). To understand whether the rescue in barrier function was driven by an increased cell viability or reduced cell death, the alamarBlue cell viability assay and ToxiLight non‐destructive cytotoxicity bioassay were performed after 48 h for all cultures. Both assays revealed no statistically significant differences between the non‐PBM and PBM‐treated groups under normoxia and hypoxia. To determine whether barrier restoration was mediated by upregulation of tight junction proteins or by other molecular mechanisms, gene and protein expression of ZO‐1 in HBMEC monocultures were analysed. RNA expression data showed no statistical difference between non‐PBM and PBM‐treated normoxia and hypoxia conditions (Fig. 1*F*). Upon ICC, ZO‐1 distribution and localisation were clearly disrupted after hypoxia compared to normoxia groups (Fig. 1*H*). Although the quantitative analysis also showed no significant difference (Fig. 1*G*), PBM appeared to reorganise ZO‐1 and improve localisation after hypoxia compared to untreated hypoxia HBMECs. Altogether, these findings reveal the therapeutic effect of PBM on HBMEC monocultures and BBB tri‐cultures following hypoxic injury.

### PBM modulates hypoxia‐induced oxidative stress and HIF‐1α signalling in a cell‐type‐specific manner

**Figure 2:**
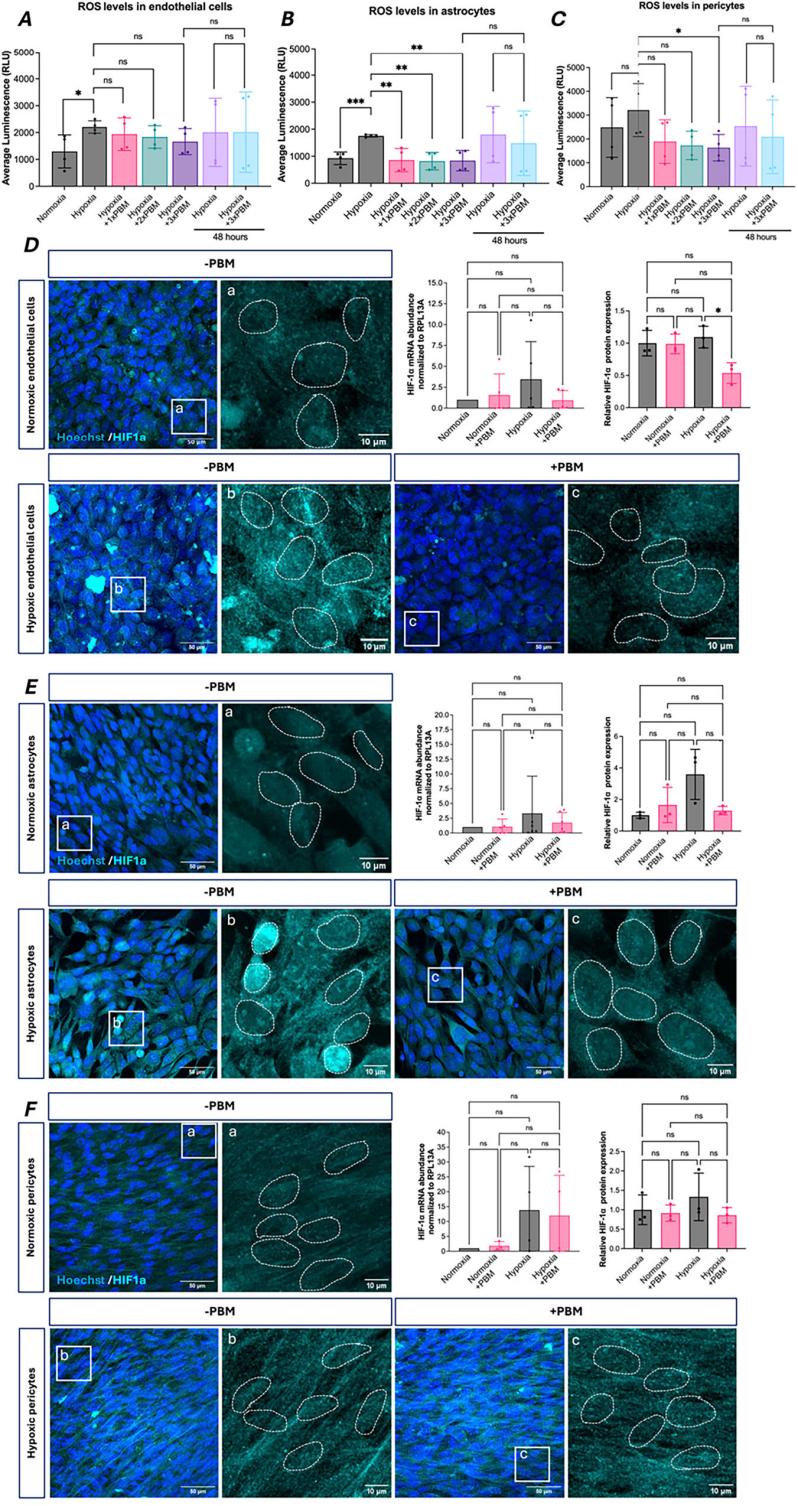
PBM attenuates oxidative stress and suppresses HIF‐1α stabilisation following hypoxic exposure ***A***–***C***, quantification of ROS levels measured by luminescence assay in endothelial cells (***A***), astrocytes (***B***) and pericytes (***C***) following hypoxia (6 h, 1% O_2_) with or without PBM treatment. PBM significantly reduced hypoxia‐induced ROS accumulation in astrocytes and pericytes, with a modest effect in endothelial cells. Statistical comparisons were performed using one‐way ANOVA with Šídák's *post hoc* correction (*P < 0.05, **P < 0.01 and ***P < 0.001); *N* = 4 independent biological replicates as independent cell cultures. Data are presented as the mean ± SD. ***D***–***F***, representative ICC images showing HIF‐1α (cyan) and nuclei (Hoechst, blue) in endothelial cells (***D***), astrocytes (***E***) and pericytes (***F***) under normoxia (–PBM), hypoxia (–PBM) and hypoxia with PBM (+PBM). Insets display higher‐magnification views illustrating nuclear localisation of HIF‐1α. The mRNA expression of HIF‐1α was normalised to RPL13A. Nuclear HIF‐1α protein levels were quantified as the mean nuclear fluorescence intensity normalised to Hoechst area and expressed relative to normoxic controls. Statistical comparisons of means from mRNA and protein expression were performed using two‐way ANOVA with Tukey's multiple comparisons test (*P < 0.05, **P < 0.01 and ***P < 0.001); *N* = 3–5 independent biological replicates as independent cell cultures. Data are presented as the mean ± SD.

Given the mechanism of action of PBM via the CCO and thus modulation of O_2_, the synthesis of ROS from the mitochondria and interrelated HIF‐1α levels after hypoxia were examined. ROS levels were measured at a ‘baseline level’ before any treatment and after one, two and three rounds of light irradiation, as well as after 48 h for each cell type. After hypoxia, HAs immediately responded to one PBM treatment with a significant halving in ROS levels, from around 1752 RLU to 857.2 RLU, which remained consistent throughout all following irradiations (*P* = 0.0061, *P* = 0.0013 and *P* = 0.003, respectively) (Fig. 2*B*). Likewise, hypoxic HVPCs show statistically significant decrease of around 50% in ROS production after three rounds of PBM (*P* = 0.0441) (Fig. 2*C*). Although PBM did not reduce ROS levels in hypoxic HBMECs (Fig. 2*A*), it markedly attenuated HIF‐1α expression, as visualised by ICC (Fig. 2*D*). To quantify HIF‐1α levels, the mean fluorescence intensity of HIF‐1α co‐localised with cell nuclei was measured, reflecting the nuclear translocation that occurs under hypoxic conditions to activate downstream signalling pathways. In hypoxic HBMECs, quantitative analysis revealed a significant reduction in nuclear HIF‐1α following PBM treatment, corresponding to an attenuation of almost three quarters after 48 h (*P* = 0.043) (Fig. 2*D*).

In astrocytes, PBM also appeared to reduce HIF‐1α levels under hypoxia, although this effect did not reach statistical significance (*P* = 0.0867) (Fig. 2*E*). By contrast, PBM had no detectable effect on HIF‐1α expression in hypoxic HVPCs (Fig. 2*F*). Consistent with the TEER measurements, PBM did not alter ROS or HIF‐1α levels under normoxic conditions, but instead modulated these responses selectively following hypoxic stress. These findings suggest that PBM acts through cell‐type‐specific mechanisms under hypoxia, with attenuation of endothelial HIF‐1α signalling representing a potential pathway contributing to improved BBB function.

### PBM increases mitochondrial respiratory capacity in endothelial cells

Following the insights that PBM altered ROS and suppressed HIF‐1α signalling, we next examined whether these effects were accompanied by changes in mitochondrial bioenergetic function in endothelial cells, astrocytes and pericytes. Cells in normoxia and hypoxia groups were treated with oligomycin, FCCP, R/A and 2‐DG after 24 and 48 h and the OCR and ECAR were measured with the Seahorse XFe 96 Extracellular Flux Analyzer.

**Figure 3:**
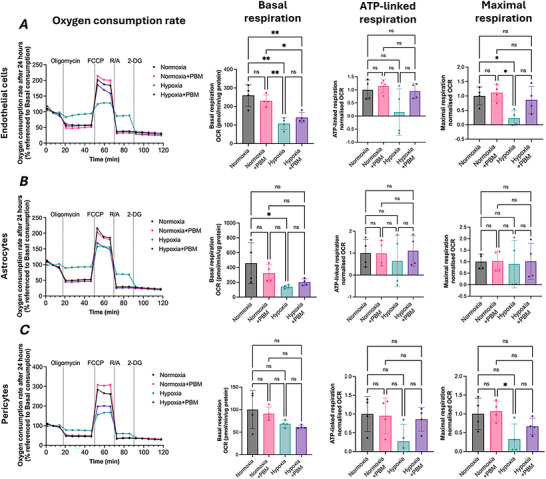
Hypoxia impacts mitochondrial respiration in endothelial cells and astrocytes, whereas PBM enhances maximal respiratory capacity in endothelial cells ***A***–***C***, mitochondrial oxygen consumption rate (OCR) profiles measured 24 h after hypoxia (6 h, 1% O_2_) in in endothelial cells (***A***), astrocytes (***B***) and pericytes (***C***), with or without PBM treatment. OCR traces are plotted as percentage change over time, normalised to protein content and baseline respiration. Sequential injections of oligomycin and FCCP were used to determine bioenergetic parameters. Basal respiration was calculated prior to oligomycin injection (pmol min^−1^ µg protein^−1^). ATP‐linked respiration was determined as the reduction in OCR following oligomycin and expressed relative to baseline OCR. Maximal respiration was calculated following FCCP treatment and normalised to OCR. Hypoxia significantly reduced mitochondrial function in endothelial cells and astrocytes, whereas PBM selectively increased maximal respiratory capacity in endothelial cells under hypoxic conditions. Statistical comparisons of means were performed using two‐way ANOVA with Tukey's multiple comparisons test (*P < 0.05, **P < 0.01 and ***P < 0.001); *N* = 4 independent biological replicates as independent cell cultures. Data are presented as the mean ± SD.

Figure 3 shows OCR dynamics following hypoxia. At 24 h post‐hypoxia, OCR profiles displayed a clear disruption compared to normoxic controls, with partial normalisation observed by 48 h. Basal respiration was significantly reduced by 58.46% in HBMECs (*P* = 0.0014) and 69.29% in HAs (*P* = 0.0427) at 24 h after hypoxia relative to normoxic cells (Fig. 3*A* and *B*). By 48 h, basal respiration had largely recovered, with no significant differences between groups.

ATP‐linked respiration, calculated as the difference between basal respiration and the oligomycin response, was markedly impaired 24 h after hypoxia. However, PBM treatment showed a trend toward restoring ATP‐linked respiration in HBMECs compared to untreated hypoxic cells, with a similar tendency observed in HAs and HVPCs. Hypoxia also significantly reduced maximal respiration in HBMECs (*P* = 0.0309), measured following FCCP injection. PBM markedly increased maximal respiratory capacity, almost restoring it toward baseline levels compared to untreated hypoxic HBMECs (*P* = 0.0753).

Proton leak and spare respiratory capacity in hypoxic HBMECs and HAs at 24 h were not significantly affected by PBM. In HVPCs, hypoxia significantly increased proton leak in both untreated (*P* = 0.0307) and PBM‐treated (*P* = 0.0297) cells relative to normoxic controls. By 48 h, OCR‐linked respiration in all cell types showed recovery following reoxygenation, with no significant differences between most groups. An exception was a physiological increase in ATP‐linked respiration in hypoxic HVPCs, observed in both untreated (*P* = 0.0023) and PBM‐treated (*P* = 0.0079) conditions compared to normoxic HVPCs.

Overall, hypoxia did not significantly alter the basal glycolytic rate (ECAR) at either 24 or 48 h compared to normoxic controls. However, at 48 h, glycolysis in HBMECs was significantly reduced below normoxic levels following hypoxia (*P* = 0.0147). Glycolytic reserve, calculated as the difference between basal glycolysis and the oligomycin response, was not significantly affected by PBM under either normoxic or hypoxic conditions at either time point.

Together, these data indicate that hypoxia primarily disrupted oxygen‐linked mitochondrial respiration in HBMECs and HAs at 24 h, with partial recovery by 48 h. Despite this recovery in overall respiratory activity, glycolytic flux remained reduced at later time points. In hypoxic endothelial cells, PBM increased maximal respiratory capacity at 24 h, indicating enhanced mitochondrial reserve and suggesting an improved ability to sustain ATP production under metabolic stress.

### PBM alters cytokine and chemokine networks in hypoxic BBB model

**Figure 4:**
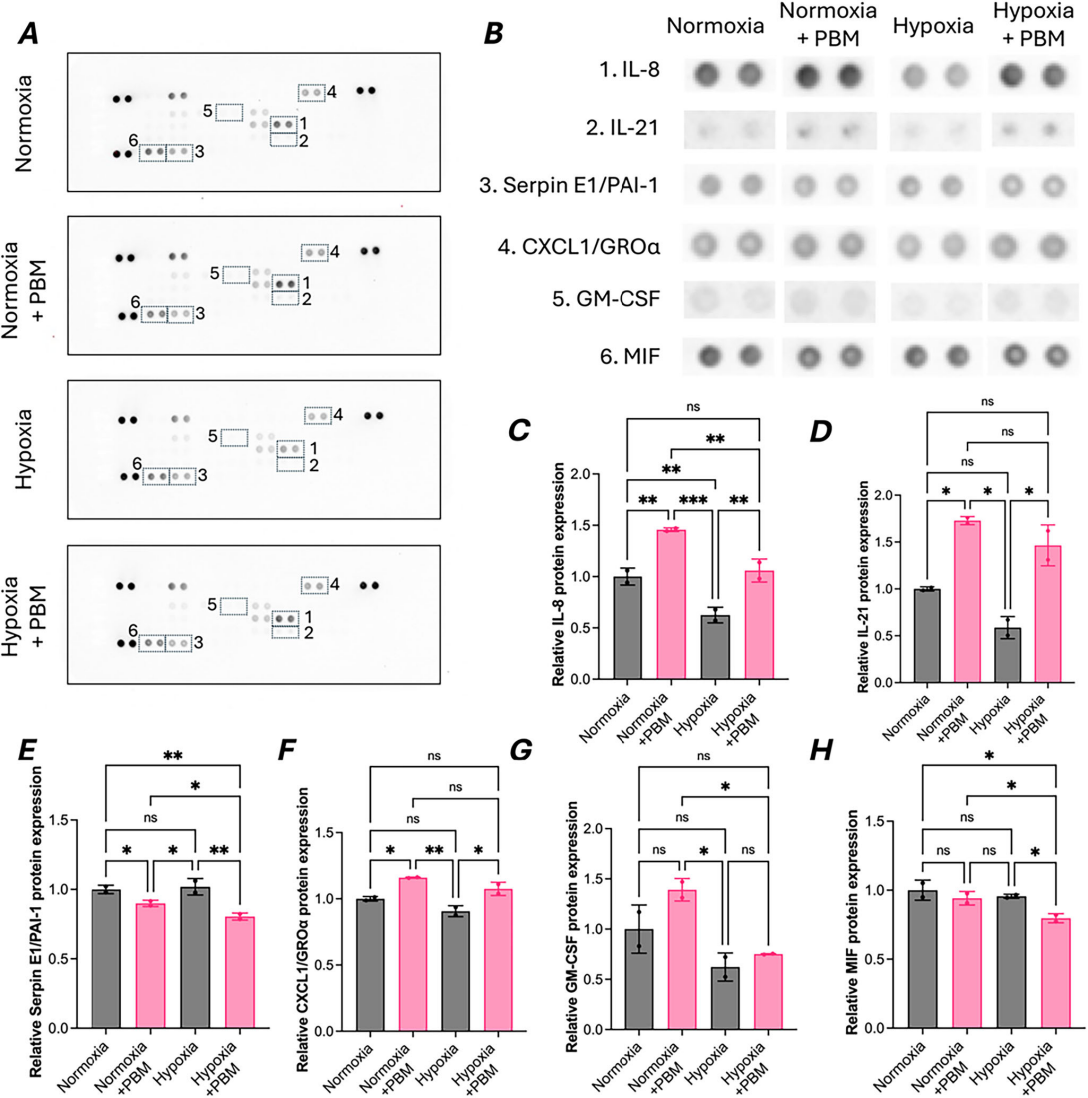
PBM differentially modulates cytokine and chemokine secretion in BBB tri‐cultures following hypoxia ***A***, representative cytokine array membranes from BBB tri‐culture supernatants under normoxia and hypoxia (6 h, 1% O_2_), with or without PBM treatment. Seventeen markers were detected above background. ***B***, quantitative summary of the six cytokines/chemokines significantly altered by PBM treatment. Spot intensities were background‐subtracted and normalised to the normoxia–PBM condition. ***C***–***H***, relative protein levels of IL‐8 (***C***), IL‐21 (***D***), Serpin E1/PAI‐1 (***E***), CXCL1/GROα (***F***), granulocyte‐macrophage colony‐stimulating factor (GM‐CSF) (***G***) and MIF (***H***). PBM significantly downregulated the pro‐thrombotic factor PAI‐1 and the pro‐inflammatory cytokine MIF, whereas it selectively modulated IL‐8, IL‐21, CXCL1 and GM‐CSF. Statistical analysis was performed using two‐way ANOVA with Tukey's multiple comparisons test (*P < 0.05, **P < 0.01 and ***P < 0.001); Data were obtained from a representative independent cell culture experiment performed with at least two technical replicates per condition and are presented as the mean ± SD.

PBM exhibits anti‐inflammatory effects and, because cells acutely respond to hypoxia by activating inflammatory pathways, it was next investigated whether PBM modulates cytokines, chemokines and acute phase proteins in the BBB. The relative protein expression of 36 human inflammatory markers was analysed in the supernatant of tri‐cultures in all four conditions at 48 h via the Proteome Profiler Human Cytokine Array (Fig. 4*A*). Six out of the 17 detected markers revealed significant differences between the four conditions (Fig. 4*B*). Under normoxic conditions, PBM significantly upregulated chemokines interleukin‐8 (IL‐8) (*P* = 0.0042) (Fig. 4*C*) and growth‐regulated oncogene‐α (CXCL1/GROα) (*P* = 0.0216) (Fig. 4*F*), as well as cytokine interleukin‐21 (IL‐21) (*P* = 0.0442) (Fig. 4*D*) compared to the untreated group. Following hypoxia, PBM also led to the upregulation of IL‐8 (*P* = 0.0049), IL‐21(*P* = 0.0266) and CXCL1/GROα (*P* = 0.0187). The plasminogen activator inhibitor‐1 (Serpin E1/PAI‐1) (Fig. 4*E*), a pro‐thrombotic protein, and migration inhibitory factor (MIF) (Fig. 4*H*), a pro‐inflammatory cytokine, were significantly downregulated upon PBM irradiation with PAI‐1 in normoxia (*P* = 0.0287) and both after hypoxia (*P* = 0.0033 and *P* = 0.0231, respectively) compared to non‐PBM groups. Collectively, these data reveal that PBM impacts the immune regulation and response independently of BBB impairment. These changes could have protective effects, such as reduced blood clot formation and promoted angiogenesis, that lead to the repair of the damaged BBB after oxidative stress.

### vWF modulation disrupts barrier integrity and abrogates the restorative effect of PBM on TEER in endothelial cells

**Figure 5:**
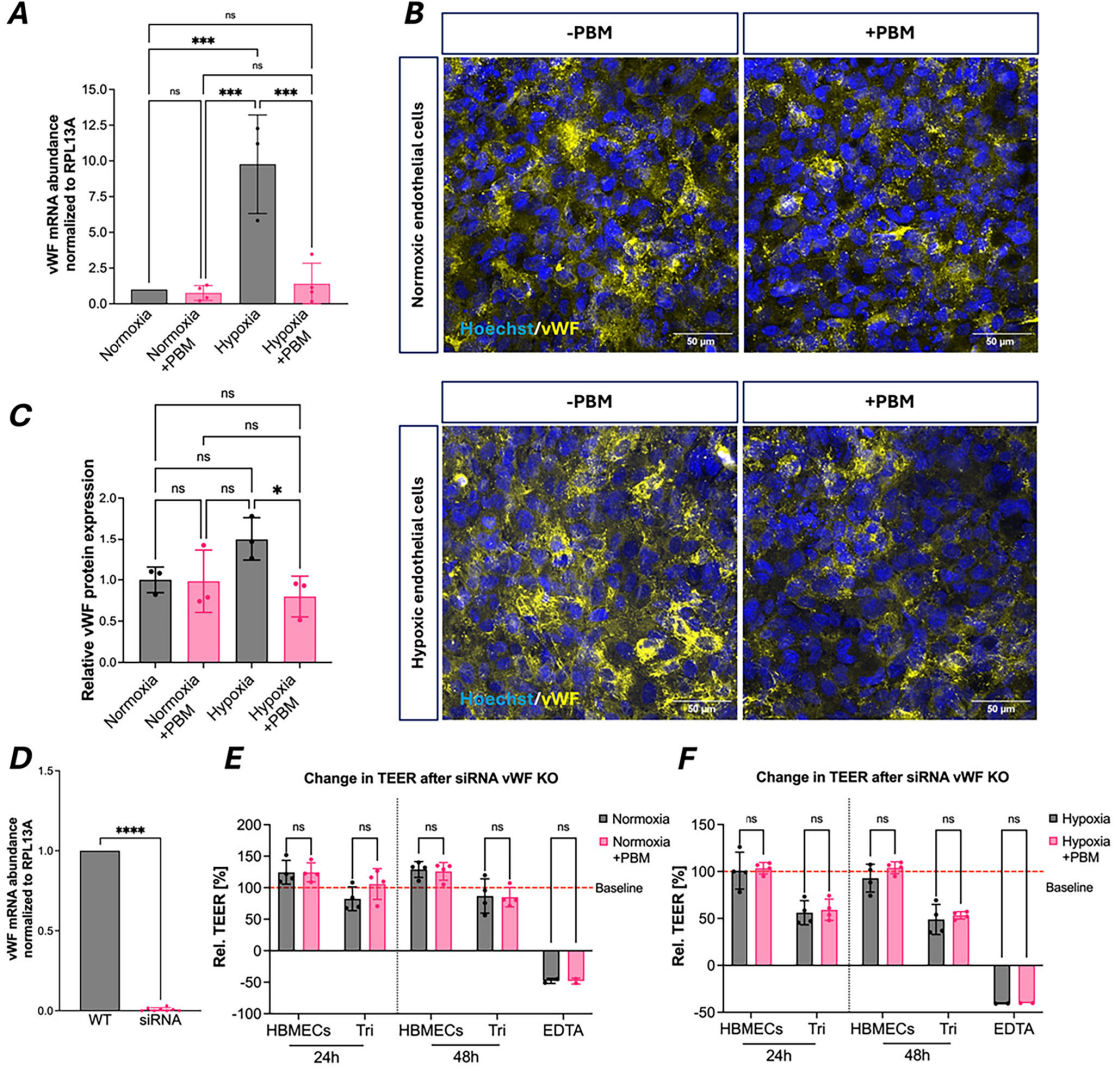
PBM restores barrier integrity through downregulation of endothelial vWF ***A***, vWF mRNA expression in HBMECs 48 h after normoxia or hypoxia (6 h, 1% O_2_), with or without PBM treatment, normalised to RPL13A. ***B***, representative ICC images showing vWF (yellow) and nuclei (Hoechst, blue) in normoxic and hypoxic endothelial cells ±PBM. ***C***, quantification of vWF protein levels, expressed as mean nuclear‐normalised fluorescence intensity relative to normoxia–PBM controls. ***D***, validation of vWF knockdown efficiency in siRNA‐transfected endothelial cells, shown as relative vWF mRNA expression normalised to RPL13A. ***E***–***F***, Relative TEER changes (%) in endothelial monocultures and BBB tri‐cultures at 24 and 48 h under normoxic (***E***) and hypoxic (***F***) conditions following vWF silencing. siRNA validation was analysed using an unpaired two‐tailed *t* test (*N* = 8 biological replicates). Statistical comparisons of means were performed using two‐way ANOVA with Tukey's multiple comparisons test (*P* < 0.05 and *P* < 0.001); *N* = 3–4 independent biological replicates as independent cell cultures. Data are presented as the mean ± SD.

The cytokine array revealed an association between upregulated IL‐8 levels and HBMECs. Weibel‐Palade bodies (WPBs), the same granules that store vWF, also serve as a reservoir for IL‐8. Given the involvement of vWF during hypoxia, its expression patterns in HBMECs were assessed. RNA expression of vWF revealed a drastic upregulation in non‐PBM‐treated HBMECs at 48 h after hypoxia (*P* = 0.0005) compared to normoxia groups (Fig. 5*A*). The same effect was also observed upon ICC after 48 h as seen in Fig. 5*B*. Most strikingly, PBM significantly alleviated this effect by 85.63% in hypoxic HBMECs, almost returning it to control levels (*P* = 0.0007), as detected by qPCR (Fig. 5*A*). Quantitative analysis of the mean intensity of vWF in ICC validated these findings on a protein level with PBM‐treated hypoxic HBMECs having reduced vWF expression compared to untreated hypoxic cells (*P* = 0.0259) (Fig. 5*C*). To evaluate whether the downregulation of vWFs is involved in improving the barrier function, HBMECs were transfected with siRNA against vWF with Lipofectamine RNAiMAX Reagent. Transfection did not significantly impact the cell count or viability (*P* = 0.0851) between wildtype HBMECs (5.380 × 10^6^ ± 1.005 cells mL^−1^) and siRNA transfected HBMECs (4.525 × 10^6^ ± 0.5443 cells mL^−1^). Moreover, an almost complete knock down of vWF was confirmed via qPCR (*P* < 0.0001) (Fig. 5*D*). Transfected HBMECs were next used to seed monocultures and tri‐cultures to assess the TEER for all four conditions at 24 and 48 h. Cultures with depleted vWF, showed no significant change in TEER between non‐PBM and PBM‐treated cultures in normoxia (Fig. 5*E*) and hypoxia (Fig. 5*F*) at both time points. Accordingly, it can be concluded that the PBM‐triggered downregulation of vWF in hypoxic HBMECs is directly linked to the improvement of barrier integrity, manifesting with an increase in TEER.

### PBM does not primarily act through classical astrocyte/pericyte reactivity markers

#### To delineate the cell‐specific contributions of perivascular astrocytes and pericytes to endothelial barrier integrity, we next examined how these cells respond to hypoxia and whether PBM modulates these responses. Astrocytes are known to undergo reactive changes under hypoxic stress, including activation of neuroinflammatory pathways marked by glial fibrillary acidic protein (GFAP) upregulation, cellular swelling and alterations in AQP4 localisation. The qPCR analysis (Fig. 6*A*) and quantitative ICC (Fig. 6*C*) showed no significant difference in AQP4 expression between normoxic and hypoxic HAs, although ICC images suggested a visual trend toward increased signal under hypoxia (Fig. 6*B*). PBM treatment did not modify this trend in hypoxic HAs compared to untreated hypoxic controls. Consistent with this, quantitative analysis of AQP4 staining intensity (Fig. 6*F*) revealed no significant difference between hypoxic and control groups

**Figure 6:**
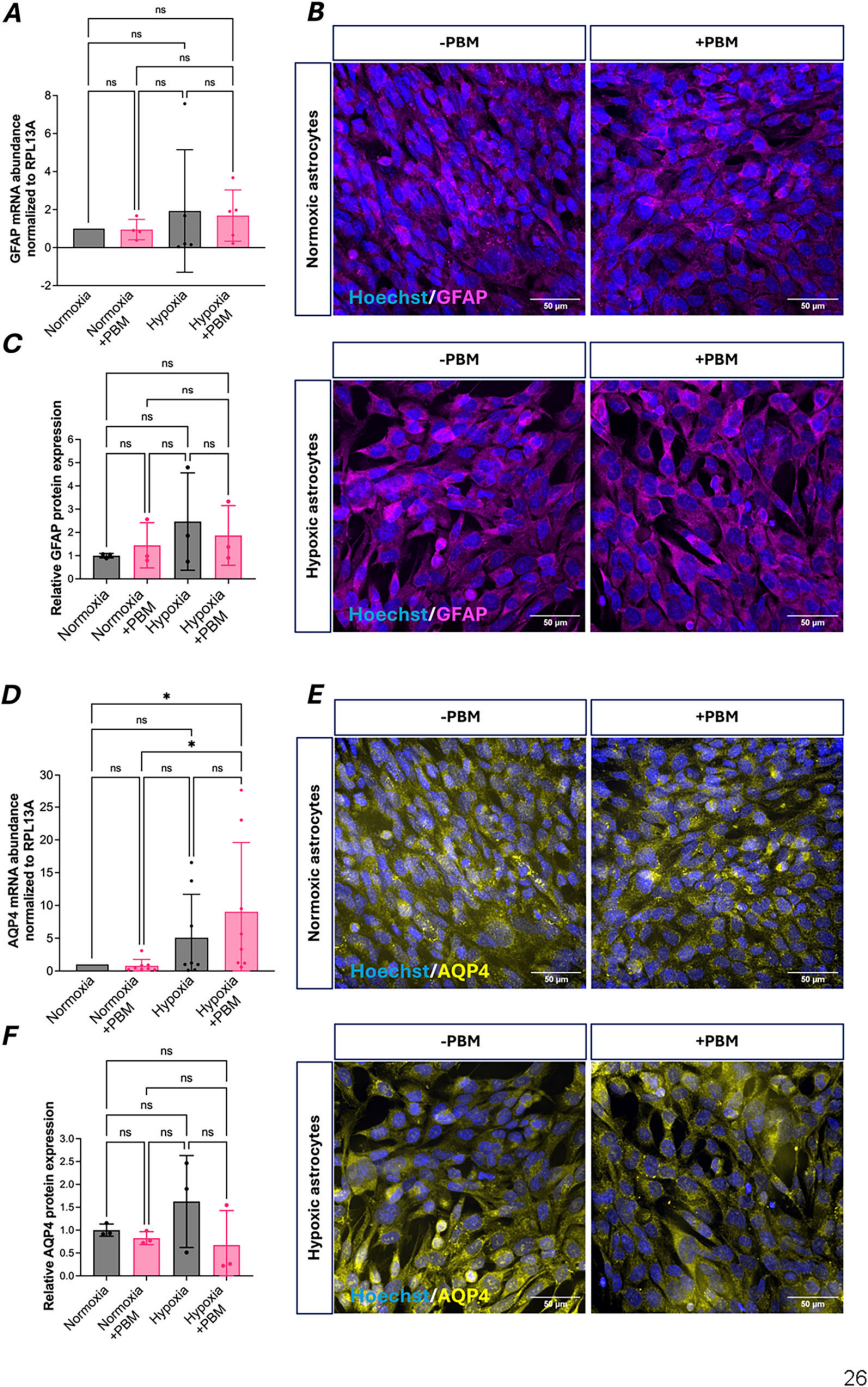
Astrocytic GFAP and AQP4 expression following hypoxia and PBM treatment ***A***, GFAP mRNA expression in astrocytes at 48 h across all experimental conditions, normalised to RPL13A. ***B***, representative ICC images showing GFAP (magenta) and nuclei (Hoechst, blue) in normoxic and hypoxic astrocytes, with and without PBM treatment. ***C***, quantification of GFAP protein expression relative to control, calculated as mean fluorescence intensity normalised to Hoechst area. ***D***, AQP4 mRNA expression in astrocytes at 48 h, normalised to RPL13A. ***E***, representative ICC images showing AQP4 (yellow) and nuclei (blue) under normoxic and hypoxic conditions ±PBM. ***F***, quantitative analysis of AQP4 protein expression relative to control, measured as mean fluorescence intensity normalised to Hoechst area. Statistical comparisons of means were performed using two‐way ANOVA with Tukey's multiple comparisons test (*P < 0.05); *N* = 3–5 independent biological replicates as independent cell cultures. Data are presented as the mean ± SD.

Interestingly, AQP4 mRNA expression in PBM‐treated hypoxic HAs was significantly increased compared to both normoxic HAs (*P* = 0.0320) and PBM‐treated normoxic cells (*P* = 0.0272). Despite this transcriptional increase, no corresponding change in protein abundance was detected across treatment groups. Notably, ICC images suggested that AQP4 signal intensity in PBM‐treated hypoxic HAs appeared lower than in untreated hypoxic cells (Fig. 6*E*), although this difference did not reach statistical significance. Overall, these data indicate that PBM does not restore BBB integrity through modulation of classical astrocytic reactivity markers because neither AQP4 nor GFAP expression was significantly altered under the conditions tested.

To date, there is limited evidence addressing how human vascular pericytes respond to PBM under normoxic or hypoxic conditions. To address this gap, we examined the expression of PDGFRβ, a canonical pericyte marker critically involved in pericyte survival, endothelial–pericyte signalling and maintenance of BBB integrity.

**Figure 7:**
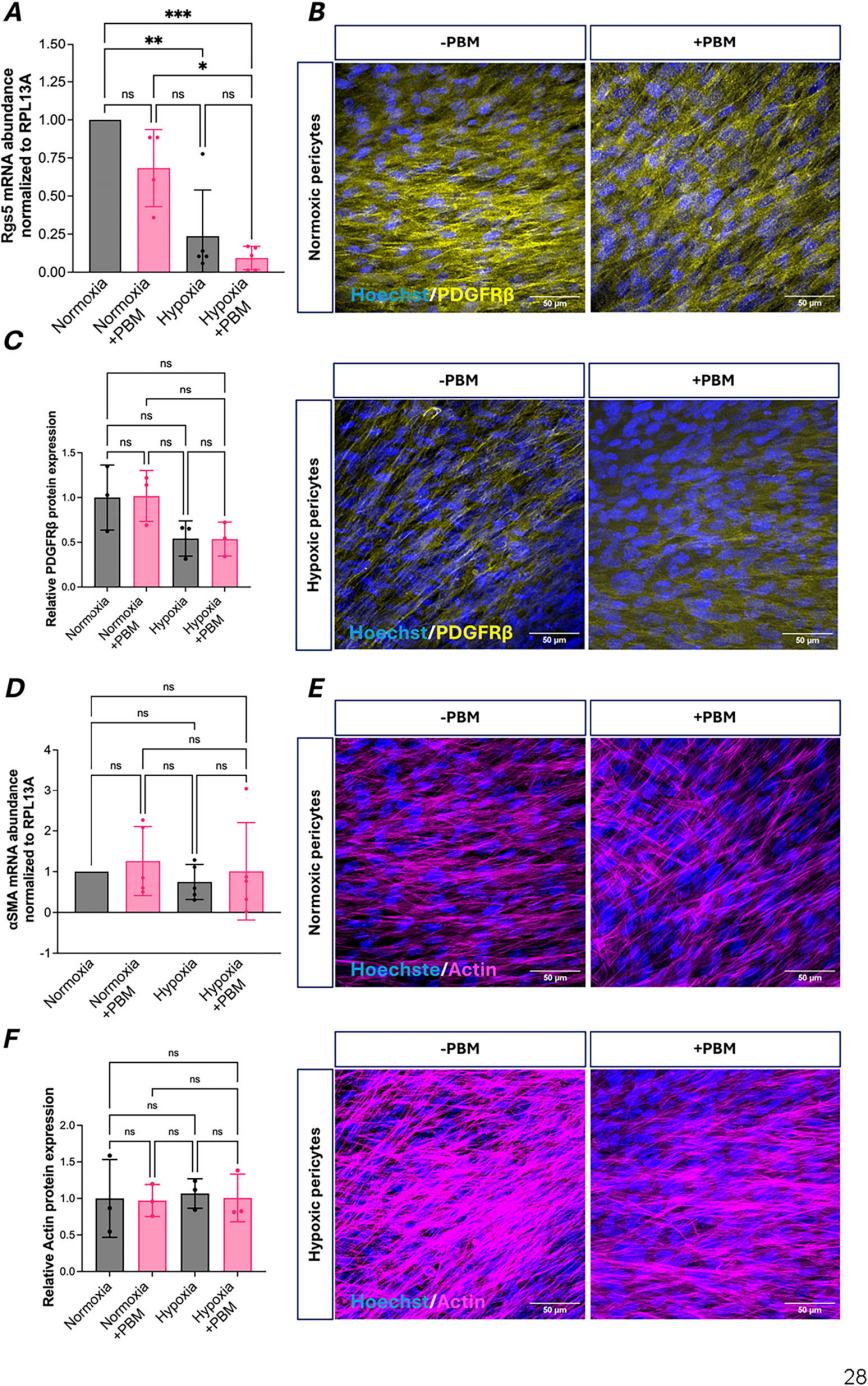
Pericyte‐specific responses: PDGFRβ, Rgs5, αSMA and actin organisation following hypoxia and PBM ***A***, Rgs5 mRNA expression in human vascular pericytes (HVPCs) at 48 h across all experimental conditions, normalised to RPL13A. ***B***, representative ICC images showing PDGFRβ (yellow) and nuclei (Hoechst, blue) in normoxic and hypoxic HVPCs, with and without PBM treatment. ***C***, quantification of PDGFRβ protein expression relative to control, calculated as mean fluorescence intensity normalised to Hoechst area. ***D***, αSMA mRNA expression in HVPCs at 48 h, normalised to RPL13A. ***E***, representative ICC images showing filamentous actin (magenta) and nuclei (blue) under normoxic and hypoxic conditions ±PBM. ***F***, quantitative analysis of actin protein expression relative to control, measured as mean fluorescence intensity normalised to Hoechst area. Statistical comparisons of means were performed using two‐way ANOVA with Tukey's multiple comparisons test (*P < 0.05); *N* = 3–5 independent biological replicates as independent cell cultures. Data are presented as the mean ± SD.

Using ICC (Fig. 7*B*), hypoxia markedly reduced PDGFRβ levels and altered its cellular localisation in HVPCs compared to normoxic controls. Quantitative analysis of mean fluorescence intensity indicated an ∼50% reduction in PDGFRβ signal under hypoxia, although this difference did not reach statistical significance (Fig. 7*C*). PBM treatment did not significantly modify PDGFRβ expression or localisation within either the normoxic or hypoxic groups.

Because PDGFRβ mRNA levels were below the detection threshold, we instead quantified expression of its upstream regulator Rgs5 under hypoxic conditions (Fig. 7*A*). By contrast to previous reports, Rgs5 expression was significantly downregulated in both untreated hypoxic cells (**P = 0.0011) and PBM‐treated hypoxic cells (***P = 0.0003) relative to untreated normoxic controls. These findings suggest that the observed reduction in PDGFRβ under hypoxia may be mediated through alternative regulatory pathways rather than canonical Rgs5‐dependent signalling.

Finally, to assess functional alterations in HVPCs across conditions, we examined cytoskeletal organisation and αSMA dynamics under hypoxia and PBM treatment. Pericyte contractility, largely governed by actin filaments and αSMA, plays a key role in regulating microvascular tone and supporting endothelial barrier stability. Although hypoxia did not significantly alter αSMA mRNA expression compared to normoxia, and PBM had no detectable transcriptional effect within groups (Fig. 7*D*), structural changes were evident at the cytoskeletal level. Immunostaining revealed that hypoxia disrupted actin alignment and promoted filament clustering and polymerisation after 48 h. By contrast, PBM‐treated HVPCs displayed a more organised actin architecture, with finer and less aggregated filaments (Fig. 7*E*). This restoration of cytoskeletal organisation may contribute to improved endothelial support and barrier integrity under hypoxic stress. Collectively, these findings highlight distinct, cell‐type‐specific responses to PBM and suggest that modulation of pericyte cytoskeletal dynamics may represent an additional mechanism through which PBM supports BBB function.

## Discussion

BBB breakdown is increasingly recognised as a central and often early feature of many CNS pathologies, including stroke, TBI and major neurodegenerative disorders, where it contributes to neuroinflammation, metabolic imbalance and progressive neuronal dysfunction. Although it remains debated whether BBB disruption is a primary driver of disease or a secondary amplifier of pathology, targeting BBB dysfunction itself probably confers a therapeutic benefit across a broad spectrum of brain conditions. In this context, the present study explored a light‐driven therapeutic strategy and its potential to restore BBB integrity following hypoxic injury. By modelling the BBB *in vitro*, we were able to directly assess barrier function and cellular physiology, leading to the identification of PBM‐mediated downregulation of vWF as a previously unrecognised mechanism contributing to barrier restoration, alongside modulation of oxidative stress and thrombo‐inflammatory signalling pathways.

A central finding of this study is that PBM significantly improves TEER in hypoxia‐exposed endothelial monocultures and BBB tri‐cultures at both 24 and 48 h, indicating sustained restoration of barrier function and warranting detailed investigation of the underlying molecular mechanisms. The platelet glycoprotein vWF is released from WPBs into the circulation and is only expressed in endothelial cells and megakaryocytes upon activation. In von Willebrand disease, a deficiency of vWF leads to the congenital bleeding disorders, including Bernard–Soulier and platelet‐type vWD (Plautz et al., 2020). Yet, high levels of vWF have been found in plasma from patients with neurological conditions, such as stroke, traumatic brain injuries and cerebral malaria (Akide Ndunge et al., 2022; Suidan et al., 2013). Overall, inflammation and endothelial damage are highly associated with vWF release, which initiates endotheliopathy and coagulopathy, both comprising pathways that significantly impair vascular health (Zeineddin et al., 2021). Moreover, various *in vivo* studies showed the direct regulatory role of vWF with respect to increasing the BBB permeability in mice upon hypoxia/reoxygenation (Suidan et al., 2013) and spontaneous intracerebral haemorrhage (Zhu et al., 2016). Furthermore, vWF induced cerebral inflammation, tight junction reorganisation, loss of pericyte coverage and neuronal injury (Zhu et al., 2016). Both studies demonstrated that genetic deletion of vWF results in a tighter BBB, characterised by reduced permeability, diminished Evans blue extravasation and lower brain water content. Beyond the prominent effect of vWF expression on barrier integrity, the structural conformation of vWF may also influence BBB function. vWF can undergo morphological transformation from a compact globular form to elongated multimeric chains under thrombo‐inflammatory conditions. These extended vWF structures facilitate leukocyte recruitment and adhesion to the endothelium, which are processes that have been associated with increased BBB permeability (Gragnano et al., 2017). In line with these findings, our data show that PBM significantly downregulates vWF expression in hypoxic endothelial cells, identifying a potential non‐invasive strategy to support restoration of BBB integrity under hypoxic stress. This is particularly important given that endothelial cells are recognised as key drivers of barrier disruption during ischaemic stroke and hypoxic injury (Knowland et al., 2014).

Unbiased profiling of secreted inflammatory mediators using a human cytokine array revealed that PBM significantly reduced the expression of PAI‐1 and macrophage MIF in BBB tri‐cultures under both normoxic and hypoxic conditions. Similarly to vWF, PAI‐1 is involved in the coagulation pathway and inhibits the serine proteases tissue‐type plasminogen activator (t‐PA) and urokinase plasminogen activator (u‐PA), thereby preventing fibrinolysis in the circulation and favouring the formation of blood clots (Vaughan et al., 2017). A recent study showed a reduction in BBB disruption and inflammation after knocking down *SerpinE1* encoding for PAI‐1 in mice subjected to ischaemic stroke compared to wild‐type animals. Furthermore, with PBM acting on two mediators of the thrombotic pathway, red and infrared light might promote the recovery of hypoxia‐related neurological conditions by reducing neuro‐inflammation via the rescue of the BBB function (Narayana et al., 2025). Consistent with this, previous studies have shown that modulation of MIF levels attenuates astrocyte activation and cytokine production both *in vitro* and *in vivo*, reduces infiltration of peripheral immune cells, and mitigates endothelial cell death and neurological deficits in murine models of perioperative ischaemic stroke (Li et al., 2023; Nasiri et al., 2020; Newell‐Rogers et al., 2020; Peng et al., 2025). Although it is unclear which BBB cell type in the tri‐culture primarily downregulated MIF upon PBM, light may not only reduce neuroinflammation, but also improve BBB permeability. Liu et al. (2018) showed that tight junction disruption in adult rat brain endothelial cells occurs upon MIF administration and that Evan blue leakage is enhanced after transient middle cerebral occlusion (tMCAo) with MIF. Treating the *in vitro* and *in vivo* models with the MIF antagonist ISO‐1 restored the ZO‐1 disruption, reduced leakage and infarct volume in tMCAo rats, and improved neurological scores (Liu et al., 2018). The PBM‐mediated downregulation of PAI‐1 and MIF could therefore additionally contribute to the improvement in barrier function and TEER after hypoxia. In addition to downregulation, PBM upregulated IL‐8 expression in tri‐cultures, which has been shown to contribute to the maintenance of endothelial cells, capillary tube organisation and angiogenesis (Li et al., 2003). Because IL‐8 is stored in WPBs and its secretion is controlled by vWF expression, the increase observed in the cytokine dot plot may be initiated by astrocytes and pericytes, highlighting the role of the cell‐to‐cell contact model (Bierings et al., 2007).

Oxidative stress is a well‐established hallmark of hypoxic injury and a key mediator of BBB disruption, contributing to endothelial dysfunction and inflammatory signalling, as well as reducing cellular viability. Hypoxia did not significantly reduce the cell viability across all cultures compared to normoxia and PBM did not alter viability or cell cytotoxicity compared to non‐PBM hypoxic cultures. Yet, upon measuring H_2_O_2 s_ynthesis after each PBM irradiation of hypoxic cells, this study showed an immediate and pronounced decrease in ROS in astrocytes and pericytes. Thus, the antioxidant nature of PBMs could be another pathway supporting the tri‐culture function and returning cells into homeostasis (Al Ahmad et al., 2012). Interestingly, OCR and ECAR data revealed that astrocytes and pericytes were less affected by hypoxia compared to endothelial cells because they show minimal differences between normoxic and hypoxic basal respiration at 24 and 48 h. Notably, canonical reactivity markers including GFAP, AQP4 and Rgs5, were not significantly upregulated following hypoxic exposure, indicating that, under the conditions tested, astrocytes and pericytes did not mount a pronounced reactive response. This may reflect intrinsic resilience to transient hypoxia and/or a rapid recovery upon reoxygenation in our model (Enström et al., 2022; Xing & Zhang, 2024). By contrast, endothelial cells exhibited clear metabolic vulnerability, with hypoxia significantly reducing basal OCR‐linked respiration. PBM appeared to selectively enhanced maximal respiratory capacity in endothelial cells at 24 h, suggesting improved mitochondrial reserve and bioenergetic flexibility. Together, these findings support the interpretation that PBM‐mediated restoration of barrier function is primarily driven by endothelial rescue rather than glial reprogramming. By directly improving endothelial metabolic competence and attenuating hypoxia‐induced dysfunction, red and near‐infrared light therapy emerges as a strategy specifically targeting vascular health, with broader implications for conditions characterised by endothelial instability and impaired neurovascular coupling. Another possible pathway involved downregulation of HIF‐1α, as detected by ICC. This link has already been discussed by Engelhardt, Al‐Ahmad et al. (2014), who reported that HIF‐1α stabilisation in hypoxia‐ or ischemia‐induced injuries is closely associated with BBB dysfunction, and that inhibition improves the barrier integrity of brain endothelial cells (Chen et al., 2009; Engelhardt et al., 2014). Thus, modulating HIF‐1α signalling, ROS levels and mitochondrial function may help preserve tight junction organisation and barrier integrity following hypoxic stress, particularly because hypoxia is known to trigger dynamic disassembly and remodelling of tight junction complexes (Knowland et al., 2014). Reduced oxygen availability, as occurs during ischaemic stroke or chronic fetal hypoxia, is a common pathological feature of many neurological and neurodegenerative disorders. Restoring BBB integrity under such conditions may therefore represent an important strategy to improve recovery after hypoxic injury, limit secondary complications and reduce long‐term neuronal damage (Engelhardt & Liebner, 2014; Herrera & González‐Candia, 2021).

Although the transwell system does not incorporate physiological shear stress or tubular geometry characteristic of 3D microvascular models, it provides a highly controlled and reproducible platform for isolating cell‐specific molecular responses and quantifying barrier integrity with precision and high throughput. For the mechanistic questions addressed here, namely the direct effects of hypoxia and PBM on endothelial‐led barrier recovery and multicellular signalling, this level of experimental control was essential. The use of immortalised human cell lines further ensured consistency across replicates, enabling robust statistical comparisons and clear attribution of molecular changes to defined cell populations.

We recognise that incorporation of flow‐based systems, primary or IPSC‐derived cells and immune components such as microglia would add additional physiological complexity and allow deeper interrogation of inflammatory cross‐talk and long‐term remodelling. These represent logical next steps building directly on the mechanistic framework established here. Similarly, although hypoxia is a broadly applicable insult, it models a convergent pathway common to stroke, TBI and chronic neurodegenerative disease, making the findings broadly relevant across CNS pathologies.

A key advantage of PBM is its non‐invasive nature, coupled with a range of beneficial cellular effects demonstrated in both *in vitro* and *in vivo* studies. However, an important unresolved challenge for clinical translation is the optimisation of dosing parameters and effective light delivery to target tissues. As light passes through the scalp, skull, meninges and brain parenchyma, scattering and absorption substantially limit penetration depth (Tedford et al., 2015). In addition, anatomical variability in skull thickness across regions such as the occipital, frontal and temporal bones significantly influences the local irradiance reaching the brain (Jagdeo et al., 2012). Consequently, careful calibration of treatment intensity, duration and anatomical targeting will be required to achieve reliable therapeutic effects and enable successful clinical translation.

Importantly, this study establishes a clear molecular link between PBM and restoration of BBB function under hypoxic stress, identifying endothelial vWF regulation and co‐ordinated oxidative and thrombo‐inflammatory modulation as actionable targets. In a therapeutic landscape where options to directly stabilise the BBB remain limited and often associated with systemic adverse effects, PBM offers a non‐invasive, mechanistically grounded strategy for vascular protection (Lemarchant et al., 2025).

## Conclusions

**Figure 8:**
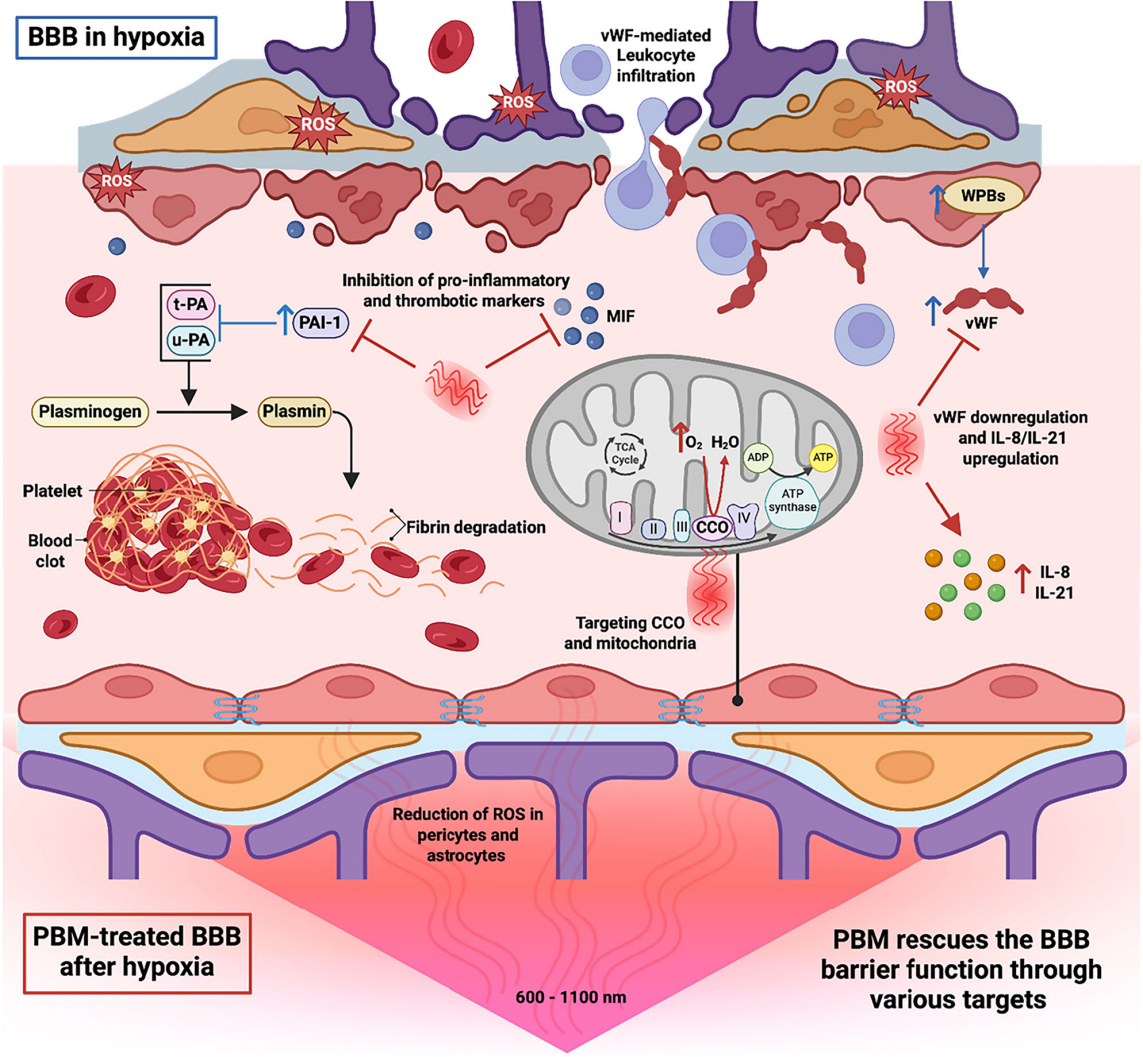
Mechanistic model of PBM‐mediated restoration of BBB integrity following hypoxia Hypoxia drives endothelial activation and thrombo‐inflammatory signalling, characterised by HIF‐1α stabilisation and vWF upregulation, resulting in barrier dysfunction. PBM reverses these processes by suppressing endothelial vWF expression and attenuating hypoxia signalling, while concurrently reducing oxidative stress in astrocytes and pericytes. This coordinated multicellular response restores barrier integrity.

In conclusion, the present study establishes a mechanistically grounded *in vitro* framework to interrogate how hypoxia disrupts BBB integrity and how targeted PBM modulates this response, as summarised in Fig. 8. Using a multicellular human BBB model, we identify endothelial cells as the principal drivers of barrier dysfunction under hypoxic stress, characterised by pronounced changes in TEER, oxidative status and secretory phenotype. By combining barrier physiology, mitochondrial bioenergetics and targeted molecular perturbation, the work moves beyond descriptive observation and identifies a discrete endothelial signalling node linking hypoxic activation to barrier failure. Importantly, PBM reproducibly restored barrier integrity and attenuated endothelial activation. Complementary genetic perturbation experiments further identified endothelial vWF as a functionally relevant mediator contributing to barrier recovery, alongside additional thrombo‐inflammatory pathways.

Although conducted in a reductionist *in vitro* system, the model enables controlled interrogation of cell‐type‐specific mechanisms (Figure 8) that are difficult to resolve *in vivo* and provides a tractable platform for future validation in primary and translational contexts. Collectively, these findings position PBM not only as a broadly cytoprotective stimulus, but also as a modulator of defined endothelial pathways that regulate BBB integrity. This work strengthens the conceptual framework linking hypoxia to endothelial activation and vascular dysfunction, and identifies actionable molecular pathways that may be targeted to restore barrier integrity in acute brain injury and chronic neurodegenerative disorders.

## Additional information

## Competing interests

The authors declare that they have no competing interests.

## Author contributions

M.M.S. conceived the study and devised the experimental strategy with L.S. M.M.S. and L.S. supervised the research. M.D. performed all the experiments and analysed the data with support from L.S. D.E.B. and N.S. provided critical knowledge to this work. M.D. assembled figures. M.D., L.S. and M.M.S. wrote the manuscript. All authors edited, read and approved the final version of the manuscript submitted for publication.

## Funding

UKRI | Medical Research Council (MRC): Mootaz M. Salman, MR/W027119/1. MMS, LS and MD are supported by a Medical Research Council Career Development Award (MR/W027119/1) and by the British Heart Foundation and the UK Dementia Research Institute (award number UK DRI‐8203) through UK DRI Ltd, principally funded by the Medical Research Council. MMS acknowledges support from the BHF Centre of Research Excellence, University of Oxford (grant code: RE/24/130024). LS acknowledges the support by the Royal Society Newton International Fellowship (NIF∖R1∖242594). Biotechnology and Biological Sciences Research Council Pioneer Award (BB/Y512874/1).

**Translational perspective**

Blood–brain barrier (BBB) dysfunction is increasingly recognised as a central contributor to neurological injury and disease progression, yet therapeutic strategies that directly target barrier repair remain limited. Our findings suggest that photobiomodulation (PBM) can restore endothelial barrier integrity after hypoxic stress by modulating defined thrombo‐inflammatory and metabolic pathways, including hypoxia‐inducible factor‐1 alpha signalling and von Willebrand factor (vWF) expression. Importantly, the data indicate that endothelial cells represent the primary responders to hypoxia within the neurovascular unit, and that targeted modulation of endothelial activation may be sufficient to stabilise barrier function. Although our work is based on an *in vitro* human BBB model, the mechanistic insights provide a rational framework for further validation in more complex systems, including organotypic models and *in vivo* settings. The identification of vWF as a functionally relevant mediator of barrier rescue highlights a pathway that could be explored pharmacologically in hypoxia‐associated conditions such as stroke, traumatic brain injury and chronic cerebrovascular insufficiency. PBM is non‐invasive and has an established safety profile in other clinical contexts. Our data therefore support the concept that carefully timed modulation of endothelial signalling could complement existing neuroprotective strategies. Future work should determine optimal dosing paradigms, depth of light penetration *in vivo* and durability of barrier rescue to evaluate whether this approach can meaningfully translate into clinical benefit.

## Supporting information




Peer Review History

**Supporting Information**: Estimated photobiomodulation dosimetry under experimental conditions.

## Data Availability

All data needed to evaluate the conclusions in the paper are present in the final publihed article and/or the Supplementary Material. This study did not generate new unique reagents.
