# Supplementary material for: Photobiomodulation restores blood–brain barrier integrity after hypoxia via endothelial von Willebrand factor modulation in a humanised tricellular transwell model
Source: J Physiol. 2026 Apr 11;604(14):6080–103. doi: 10.1113/JP291064 (PMC13370692; doi:10.1113/JP291064)
Supplement: Supplementary file 1 — Peer Review History [file TJP-604-6080-s002.pdf]

The Journal of Physiology

# Photobiomodulation restores blood-brain barrier integrity after hypoxia via endothelial vWF modulation in a humanised tricellular transwell model

Mirriam Domocos, Denis E Bragin, Nagesh C Shanbhag, Luise Schlotterose, and Mootaz M. Salman
**DOI: 10.1113/JP291064**

*Corresponding author(s): Mootaz Salman (mootaz.salman@dpag.ox.ac.uk)*

*The following individual(s) involved in review of this submission have agreed to reveal their identity: Matthew Conner (Referee #2)*

## Review Timeline:

| | |
|---|---|
| Submission Date: | 13-Feb-2026 |
| Editorial Decision: | 02-Mar-2026 |
| Revision Received: | 15-Mar-2026 |
| Accepted: | 19-Mar-2026 |

*Senior Editor: Nathan Schoppa*

*Reviewing Editor: Valentina Mosienko*

## Transaction Report:

(Note: With the exception of the correction of typographical or spelling errors that could be a source of ambiguity, letters and reports are not edited. Depending on transfer agreements, referee reports obtained elsewhere may or may not be included in this compilation. Referee reports are anonymous unless the Referee chooses to sign their reports.)

Dear Dr Schlotterose,

Re: JP-RP-2026-291064 "Light-driven repair: Photobiomodulation promotes blood-brain barrier recovery following hypoxic injury" by Mootaz M. Salman, Mirriam Domocos, Denis E Bragin, Nagesh C Shanbhag, and Luise Schlotterose

Thank you for submitting your manuscript to The Journal of Physiology. It has been assessed by a Reviewing Editor and by 2 expert referees and we are pleased to tell you that it is acceptable for publication following satisfactory revision.

The referee reports are copied at the end of this email.

Please address all the points raised and incorporate all requested revisions or explain in your Response to Referees why a change has not been made. We hope you will find the comments helpful and that you will be able to return your revised manuscript within 4 weeks. If you require longer than this, please contact journal staff: jp@physoc.org.

Your revised manuscript should be submitted online using the link in your Author Tasks Link Not Available. This link is accessible via your account as Corresponding Author; it is not available to your co-authors. If this presents a problem, please contact journal staff (jp@physoc.org). Image files from the previous version are retained on the system. Please ensure you replace or remove any files that are being revised.

If you do not wish to submit a revised version of your manuscript, you must inform our journal staff (jp@physoc.org) or reply to this email to request withdrawal. Please note that a manuscript must be formally withdrawn from the peer review process at one journal before it may be submitted to another journal.

TRANSPARENT PEER REVIEW POLICY: To improve the transparency of its peer review process The Journal of Physiology publishes online, as supporting information, the peer review history of all articles accepted for publication. Readers will have access to decision letters, including Editors' comments and referee reports, for each version of the manuscript, as well as any author responses to peer review comments. Referees can decide whether or not they wish to be named on the peer review history document.

ABSTRACT FIGURES: Authors are expected to use The Journal's premium BioRender account to create/redraw their Abstract Figures. Information on how to access this account is here: https://physoc.onlinelibrary.wiley.com/journal/14697793/biorender-access.

This will enable Authors to create and download high-resolution figures. If authors have used the free BioRender service, they can use the instructions provided in the link above to download a high-resolution version suitable for publication.

The link provided should only be used for the purposes of this submission. Authors will be charged for figures created on this account if they are not related to this manuscript submission.

LANGUAGE EDITING AND SUPPORT FOR PUBLICATION: If you would like help with English language editing, or other article preparation support, Wiley Editing Services offers expert help, including English Language Editing, as well as translation, manuscript formatting, and figure formatting at www.wileyauthors.com/eeo/preparation. You can also find resources for Preparing Your Article for general guidance about writing and preparing your manuscript at www.wileyauthors.com/eeo/prepresources.

RIGOUR AND REPRODUCIBILITY: We recommend authors consult The Journal's Rigour and Reproducibility requirements to ensure that their manuscript is suitable for publication: https://physoc.onlinelibrary.wiley.com/pb-assets/hub-assets/physoc/documents/TJP-Rigour-and-Reproducibility-Requirements.pdf

REVISION CHECKLIST:

Check that your Methods section conforms to journal policy: https://jp.msubmit.net/cgi-bin/main.plex?form_type=display_requirements#methods

Check that data presented conforms to the statistics policy: https://jp.msubmit.net/cgi-bin/main.plex?form_type=display_requirements#statistics

Upload a full Response to Referees file. To create your 'Response to Referees' copy all the reports, including any comments from the Senior and Reviewing Editors, into a Microsoft Word, or similar, file and respond to each point, using font or background colour to distinguish comments and responses and upload as the required file type.

Please upload two versions of your manuscript text: one with all relevant changes highlighted and one clean version with no changes tracked. The manuscript file should include all tables and figure legends, but each figure/graph should be uploaded as separate, high-resolution files. The journal is now integrated with Wiley's Image Checking service. For further details, see: https://www.wiley.com/en-us/network/publishing/research-publishing/trending-stories/upholding-image-integrity-wileys-image-screening-service

You may also upload:

- 'Potential Cover Art' for consideration as the issue's cover image
- Appropriate Supporting Information (Video, audio or data set: see https://jp.msubmit.net/cgi-bin/main.plex?form_type=display_requirements#supp).

We look forward to receiving your revised submission.

If you have any queries, please reply to this email and we will be pleased to advise.

Yours sincerely,

Nathan Schoppa
Senior Editor
The Journal of Physiology

----------------

REQUIRED ITEMS

- Author photo and profile. First or joint first authors are asked to provide a short biography (no more than 100 words for one author or 150 words in total for joint first authors) and a portrait photograph. These should be uploaded and clearly labelled together in a Word document with the revised version of the manuscript. See Information for Authors for further details.

- The contact information for the person responsible for 'Research Governance' at your institution needs to be provided. This includes their name and an institutional email address. Please ensure the contact is not an author on this paper and provide an alternate contact if necessary, or confirm in the submission form that the author whose email was provided has sole responsibility for research governance. This is the person who is responsible for regulations, principles and standards of good practice in research carried out at the institution, for instance the ethical treatment of animals, the keeping of proper experimental records or the reporting of results.

- The reference list must be in alphabetical order, rather than numbered, to comply with our Journal format.

- Please upload separate high-quality figure files via the submission form.

- Your paper contains Supporting Information of a type that we no longer publish, including supplementary tables and figures. Any information essential to an understanding of the paper must be included as part of the main manuscript and figures. The only Supporting Information that we publish are video and audio, 3D structures, program codes and large data files. Your revised paper will be returned to you if it does not adhere to our Supporting Information Guidelines.

- Papers must comply with the Statistics Policy: https://jp.msubmit.net/cgi-bin/main.plex?form_type=display_requirements#statistics.

In summary:

- If n {less than or equal to} 30, all data points must be plotted in the figure in a way that reveals their range and distribution. A bar graph with data points overlaid, a box and whisker plot or a violin plot (preferably with data points included) are acceptable formats.

- If n > 30, then the entire raw dataset must be made available either as supporting information, or hosted on a not-for-profit repository, e.g. FigShare, with access details provided in the manuscript.

- 'n' clearly defined (e.g. x cells from y slices in z animals) in the Methods. Authors should be mindful of pseudoreplication.

- All relevant 'n' values must be clearly stated in the main text, figures and tables.

- The most appropriate summary statistic (e.g. mean or median and standard deviation) must be used. Standard Error of the Mean (SEM) alone is not permitted.

- Exact p values must be stated. Authors must not use 'greater than' or 'less than'. Exact p values must be stated to three significant figures even when 'no statistical significance' is claimed.

- Please include an Abstract Figure file and an Abstract Figure legend. An appropriate figure legend, which should not exceed 150 words in length, should be included in the main manuscript file. The Abstract Figure is a piece of artwork designed to give readers an immediate understanding of the research and should summarise the main conclusions. If possible, the image should be easily 'readable' from left to right or top to bottom. It should show the physiological relevance of the manuscript so readers can assess the importance and content of its findings. Abstract Figures should not merely recapitulate other figures in the manuscript. Please try to keep the diagram as simple as possible and without superfluous information that may distract from the main conclusion(s). Abstract Figures must be provided by authors no later than the revised manuscript stage and should be uploaded as a separate file during online submission labelled as File Type 'Abstract Figure'. Please also ensure that you include the figure legend in the main article file. All Abstract Figures should be created using BioRender. Authors should use The Journal's premium BioRender account to export high-resolution images. Details on how to use and access the premium account are included as part of this email.

----------------

EDITOR COMMENTS

Reviewing Editor:

Comments for Authors to ensure the paper complies with the Statistics Policy (Required):
Please, make sure that the manuscript complies with the Journal's statistical policy - specifically, please, make sure that all graphs data are displayed as mean +/- SD (not SEM), precise values are given (either in the text or figure legend).

Comments to the Author (Required):
This is an excellent and comprehensive study that clearly identifies the cellular and molecular mechanisms underlying the therapeutic effects of photobiomodulation on the blood-brain barrier. The manuscript has been favorably evaluated by two expert reviewers, both of whom raised only minor concerns primarily related to clarification to the text. Please address these points carefully during the revision process.

In the revised version, please ensure that two-way ANOVA is performed for datasets involving two independent variables. For example, this applies to Figures 1F and 1G (variable 1: normoxia vs. hypoxia; variable 2: PBM vs. no stimulation), the graphs presented in Figure 2, all graphs in Figures 3 and 4, and Figure 5A and 5C.

As the journal discourages extensive use of supplementary material, please consider incorporating supplementary data into the main manuscript wherever feasible, either within the main text or integrated into the figures.

Please also see 'Required Items' above.

Senior Editor:

Comments for Authors to ensure the paper complies with the Statistics Policy (Required):
Please report SDs rather than SEMs and report precise p-values (unless $p < 0.001$).

Comments to the Author:
Thank you for submitting your manuscript to Journal of Physiology. The study has been reviewed by two expert referees and a reviewing editor, who all appreciated its mechanistic depth and likely impact toward the understanding of how photobiomodulation can restore blood-brain barrier integrity. The work was also considered to be quite nicely presented. Congratulations on an excellent study!

The manuscript is considered to be potentially acceptable for publication pending your addressing the minor concerns that were raised by the referees about clarifications in the text. Also, please address the concerns raised by the reviewing editor around statistics and reporting of SEMs, to which I will add that you will also need to report precise p-values (per Journal policy). Also, the Journal does not allow Supplementary Figures. We would encourage you to incorporate the most important results into the main figures and/or text. The Journal does allow the inclusion of Appendix items that are part of the final

manuscript, and some of the results may be presented there. Finally, we note that the quality of some of the graphs and images appears somewhat reduced in the merged PDF, which can occur during automatic file generation. With submission of the revision, we encourage you to resubmit your figures as separate high-resolution files. We look forward to receiving your revision.

-----------------

REFEREE COMMENTS

Referee #1:

This manuscript presents an elegant and mechanistically insightful investigation into the effects of photobiomodulation (PBM) on BBB integrity following hypoxic injury in a humanized tricellular transwell model. The study stands out for its depth of experimentation, clarity of mechanistic focus, and translational relevance.

The authors combine functional barrier readouts (automated TEER via CellZScope 2), mitochondrial bioenergetics (Seahorse profiling), redox signaling, HIF-1α modulation, and targeted suppression of endothelial vWF to construct a coherent mechanistic framework linking hypoxia-induced endothelial dysfunction to barrier breakdown, and importantly, to PBM-mediated rescue. The use of CellZScope 2 is particularly commendable, as it allows continuous, automated impedance monitoring without the perturbations often introduced by manual EVOM measurements. This strengthens the reliability of the barrier integrity data and enhances confidence in the temporal dynamics reported.

Up to my knowledge, this is the first mechanistic study demonstrating that PBM restores BBB function through modulation of endothelial thrombo-inflammatory signaling, specifically vWF, in a humanized tricellular transwell system. Given that PBM is already under clinical investigation, this mechanistic anchoring is both timely and highly impactful. The work therefore carries genuine translational weight, moving beyond descriptive therapeutic claims toward molecular causality.

The proteome array work is another strength. The unbiased screening approach strengthens the conceptual narrative and allows the authors to pivot from discovery to mechanistic targeting. This elevates the study beyond candidate-driven approaches and provides a robust foundation for future in vivo validation.

The authors appropriately acknowledge the limitations of immortalized cell systems and suggest iPSC-derived or primary models as future extensions. While such systems may provide incremental physiological refinement, the tricellular transwell configuration used here is highly appropriate for BBB mechanisms in a scable approach that's needed to study mechanisms of barrier regulation. For the mechanistic questions addressed in this study, the model is well chosen and experimentally fit-for-purpose.

Suggestions to strengthen the manuscript further:

Introduction: A concise paragraph early in the Introduction summarizing established mitochondrial/cytochrome c oxidase-mediated PBM mechanisms would help orient readers unfamiliar with the photobiology field. For example, the role of cytochrome c oxidase as a primary photoacceptor (Karu, 1999; Wong-Riley et al., 2005), mitochondrial membrane potential modulation (Rojas & Gonzalez-Lima, 2011), and redox-sensitive transcriptional changes (Hamblin, 2017; Salehpour et al., 2018) could be briefly referenced. Additionally, citing current clinical or translational PBM work targeting neurological disorders (e.g., Berman et al., 2017; Chao, 2019; Salehpour & Hamblin, 2020) would contextualize the clinical relevance.

Discussion: Dosimetry and Translation: A short discussion of light penetration constraints and dosing considerations for in vivo application would enhance the translational framing. Even a brief acknowledgment of tissue scattering and skull attenuation (Jagdeo et al., 2012; Tedford et al., 2015) would demonstrate awareness of clinical implementation challenges.

Title: I recommend dropping the phrase "light to repair," which feels promotional rather than mechanistic. A more precise and physiology-centered title would be:

Photobiomodulation restores blood-brain barrier integrity after hypoxia via endothelial vWF modulation in a humanized tricellular transwell model

To conclude, this is a carefully executed mechanistic study with clear physiological and translational implications. The integration of endothelial bioenergetics, thrombo-inflammatory signaling, and barrier physiology represents a meaningful advance in the PBM field. I support publication after minor clarifications as outlined above.

Referee #2:

This is a novel, impactful and conceptually strong manuscript that provides mechanistic understanding of how photobiomodulation (PBM) restores blood-brain barrier (BBB) integrity following acute hypoxic injury and through which cell type-specific mechanisms. The authors are commended for advancing the work beyond phenomenology toward molecular resolution, offering one of the first analyses of PBM effects on defined endothelial and glial pathways in a humanised BBB model. The identification of endothelial vWF as a target for barrier repair, combined with current clinical investigations of PBM in neurological disease, underscores its strong translational relevance.

The study uses a human tricellular transwell BBB model to show that repeated PBM rescues hypoxia induced loss of TEER, linked mechanistically to downregulation of endothelial von Willebrand factor (vWF), reduced HIF 1α signalling, and cell type-specific redox and bioenergetic effects. TEER measurements, mitochondrial bioenergetics, oxidative stress readouts, cytokine profiling, and targeted genetic manipulation are employed to build a coherent mechanistic narrative centered on endothelial thrombo-inflammatory signalling and vWF modulation and thus the study constitutes a meaningful advance with significant translational implications.

The manuscript is suitable for publication after minor textual refinements that will further strengthen clarity and positioning.

Major strengths

The mechanistic depth is a clear highlight. The identification of endothelial vWF as a mediator of barrier rescue following PBM is novel and conceptually compelling. The use of siRNA to partially recapitulate PBM's effect meaningfully strengthens the causal argument.

The integration of metabolism and barrier biology is well articulated and biologically plausible, consistent with emerging literature on endothelial bioenergetics in barrier regulation, including work by Zhao et al. (Nature Metabolism, 2020) and Shetty et al. (Journal of Cerebral Blood Flow & Metabolism, 2022).

The human-relevant multicellular configuration appropriately captures endothelial-pericyte-astrocyte interactions and represents a strong fit-for-purpose platform for mechanistic interrogation of hypoxia-driven BBB dysfunction.

Minor:

Figure 1 Legend: ensure spacing is consistent after the letters e.g. "(G)Quantification" no space, "(B) Normoxic controls" has a space.

The Introduction could more explicitly state, in the final paragraph, the central hypothesis being tested, namely that PBM restores BBB integrity through defined endothelial thrombo-inflammatory signalling pathways in a humanised model. Sharpening this framing would elevate the conceptual clarity.

The Introduction would benefit from clearer positioning of PBM within current mechanistic debates. A brief summary of proposed mechanisms, particularly mitochondrial cytochrome c oxidase modulation and nitric oxide photodissociation (e.g., Hamblin, Photochemistry and Photobiology, 2018; Poyton and Ball, PNAS, 2011), would anchor the study more firmly within the existing literature.

The Discussion is strong but could benefit from emphasising that vWF modulation represents one important pathway among potentially several mechanisms contributing to PBM-induced barrier recovery. Briefly positioning the findings within the broader context of hypoxia-induced BBB disruption in stroke and perinatal hypoxia models (e.g., Knowland et al., Neuron, 2014; Engelhardt and Liebner, Nature Reviews Neuroscience, 2014) would further enhance balance and context. It would also be useful to include a short description of light penetration and tissue attenuation constraints (e.g., Jacques, Physics in Medicine & Biology, 2013) would strengthen the translational perspective.

The Supplementary material is comprehensive but could be streamlined. Consolidating some validation panels into written results may help without reducing transparency.

---

END OF COMMENTS

Dear Professor Nathan Schoppa and Dr Valentina Mosienko,

Thank you for the opportunity to revise our manuscript now entitled:

Photobiomodulation restores blood–brain barrier integrity after hypoxia via endothelial vWF modulation in a humanised tricellular transwell model.

We were delighted by the very positive and encouraging evaluation of our work and are grateful to you and the reviewers for the careful review, thoughtful scrutiny, and constructive feedback provided throughout the process. We appreciate the reviewers' recognition of the mechanistic depth, physiological relevance, and translational potential of the study.

We have carefully revised the manuscript in response to all comments. For clarity, all modifications in the revised manuscript are highlighted in blue. The main changes are summarised below.

Summary of revisions:

• Statistics updated throughout the manuscript to comply fully with the Journal of Physiology statistical policy, including use of SD instead of SEM, reporting of exact p values, and application of two-way ANOVA where two independent variables are present.

• Figures updated to display all individual data points as required by the journal's statistical guidelines.

• Exact n values are now clearly defined in the Methods, figure legends, and Results text.

• Reference list reformatted into alphabetical order to comply with journal formatting requirements.

• All references checked and corrected for consistency and accuracy.

• The Introduction has been expanded to include a concise overview of established PBM mechanisms, including cytochrome c oxidase signalling, mitochondrial membrane potential modulation, nitric oxide photodissociation, and redox-sensitive transcriptional responses.

• The final paragraph of the Introduction has been sharpened to explicitly state the central hypothesis that PBM restores BBB integrity through defined endothelial thrombo-inflammatory signalling pathways in a humanised model.

• The Discussion has been revised to further contextualise PBM within current mechanistic debates and to clarify that vWF represents one important pathway among several mechanisms contributing to barrier recovery.

• Additional references have been incorporated where suggested by the reviewers.

• A short section discussing light penetration and dosimetry considerations for PBM translation has been added.

• Supplementary figures and tables have been removed in accordance with journal policy, and the most relevant data have been integrated into the main manuscript and figures.

• All figures have been incorporated as high-resolution files.

• An Abstract Figure and legend have been added as requested.

• Minor textual clarifications and figure legend corrections have been implemented throughout the manuscript.

We believe these revisions have strengthened the clarity, statistical rigour, and contextual framing of the manuscript, and we hope the revised version meets the requirements for publication in The Journal of Physiology.

Thank you again for your thoughtful editorial guidance and for the constructive comments provided by the reviewers.

Sincerely,

Mootaz M. Salman
on behalf of all authors

Dear Dr Salman,

Re: JP-RP-2026-291064R1 "**Photobiomodulation restores blood-brain barrier integrity after hypoxia via endothelial vWF modulation in a humanised tricellular transwell model**" by Mirriam Domocos, Denis E Bragin, Nagesh C Shanbhag, Luise Schlotterose, and Mootaz M. Salman

We are pleased to tell you that your paper has been accepted for publication in The Journal of Physiology.

***IMPORTANT***
Please see comments below for some minor amendments that should be made at proof stage.

The last Word (or similar) version of the manuscript provided will be used by the Production Editor to prepare your proof. When this is ready you will receive an email containing a link to Wiley's Online Proofing System. The proof should be thoroughly checked and corrected as promptly as possible.

Authors should note that it is too late at this point to offer corrections prior to proofreading. Major corrections at proof stage, such as changes to figures, will be referred to the Editors for approval before they can be incorporated. Only minor changes, such as to style and consistency, should be made at proof stage. Changes that need to be made after proof stage will usually require a formal correction notice.

All queries at proof stage should be sent to: TJP@wiley.com.

Following copyediting and typesetting the accepted version will be published online.

Yours sincerely,

Nathan Schoppa
Senior Editor
The Journal of Physiology

IMPORTANT POINTS TO NOTE FOLLOWING ACCEPTANCE OF YOUR PAPER:

• IMPORTANT NOTICE ABOUT OPEN ACCESS: To assist authors whose funding agencies mandate immediate public access to published research findings, The Journal of Physiology allows authors to pay an Open Access (OA) fee to have their papers made freely available immediately on publication.

The Corresponding Author will receive an email from Wiley with details on how to register or log in to Wiley Authors where you will be able to place an order.

• You can check if your funder or institution has a Wiley Open Access Account here:
https://authors.wiley.com/author-resources/Journal-Authors/open-access/author-compliance-tool.html

• TRANSPARENT PEER REVIEW POLICY: To improve the transparency of its peer review process, The Journal of Physiology publishes online as supporting information the peer review history of all articles accepted for publication. Readers will have access to decision letters, including Editors' comments and referee reports, for each version of the manuscript as well as any author responses to peer review comments. Referees can decide whether or not they wish to be named on the peer review history document.

• You can help your research get the attention it deserves! Check out Wiley's free Promotion Guide for best-practice recommendations for promoting your work at: www.wileyauthors.com/eeo/guide. You can learn more about Wiley Editing Services which offers professional video, design, and writing services to create shareable video abstracts, infographics, conference posters, lay summaries, and research news stories for your research at: www.wileyauthors.com/eeo/promotion.

• If you would like to receive our 'Research Roundup', a monthly newsletter highlighting the cutting-edge research published in The Physiological Society's family of journals (The Journal of Physiology, Experimental Physiology, Physiological Reports, The Journal of Nutritional Physiology and The Journal of Precision Medicine: Health and Disease), please click this link, fill in your name and email address and select 'Research Roundup':
https://www.physoc.org/journals-and-media/membernews

----------------

EDITOR COMMENTS

Reviewing Editor:

Thank you for submitting the revised version of your manuscript and for thoughtfully addressing the reviewers' comments. I have no further concerns.

One minor point: please update the statistics section of the Methods to state that two-way ANOVA was performed on the relevant datasets.

Senior Editor:

Thank you for submitting your revised manuscript to The Journal of Physiology. We are pleased to report that the study is now acceptable for publication. We appreciate the additional statistical analyses that you performed and the additional changes to the text.

We noted a couple of very minor points in the Statistics part of the Methods section that can be corrected in the proofs stage. Please clarify that you used two-way ANOVA (not one-way) on relevant datasets. Also, the Methods still indicates that you report SEMs, even though you now report SDs. I also noted that SEM is indicated in the Abbreviations section.

-----------------
